# Tracking spending on malaria by source in 106 countries, 2000–16: an economic modelling study

**DOI:** 10.1016/S1473-3099(19)30165-3

**Published:** 2019-07

**Authors:** Annie Haakenstad, Anton Connor Harle, Golsum Tsakalos, Angela E Micah, Tianchan Tao, Mina Anjomshoa, Jessica Cohen, Nancy Fullman, Simon I Hay, Tomislav Mestrovic, Shafiu Mohammed, Seyyed Meysam Mousavi, Molly R Nixon, David Pigott, Khanh Tran, Christopher J L Murray, Joseph L Dieleman

**Affiliations:** a Harvard T H Chan School of Public Health, Boston, MA, USA; b Institute for Health Metrics and Evaluation, Seattle, WA, USA; c Rafsanjan University of Medical Sciences Social Determinants of Health Research Center, Rafsanjan, Iran; d Department of Health Management and Economics, Tehran University of Medical Sciences, Tehran, Iran; e Dr Zora Profozic Polyclinic Clinical Microbiology and Parasitology Unit, Zagreb, Croatia; f University Centre Varazdin, Varazdin, Croatia; g Ahmadu Bello University, Zaria, Nigeria; h Heidelberg University Institute of Public Health, Heidelberg, Germany; i Tehran University of Medical Sciences Department of Health Management and Economics, Tehran, Iran; j University of Auckland Department of Molecular Medicine and Pathology, Auckland, New Zealand; k Military Medical University Department of Clinical Hematology and Toxicology, Hanoi, Vietnam

## Abstract

**Background:**

Sustaining achievements in malaria control and making progress toward malaria elimination requires coordinated funding. We estimated domestic malaria spending by source in 106 countries that were malaria-endemic in 2000–16 or became malaria-free after 2000.

**Methods:**

We collected 36 038 datapoints reporting government, out-of-pocket (OOP), and prepaid private malaria spending, as well as malaria treatment-seeking, costs of patient care, and drug prices. We estimated government spending on patient care for malaria, which was added to government spending by national malaria control programmes. For OOP malaria spending, we used data reported in National Health Accounts and estimated OOP spending on treatment. Spatiotemporal Gaussian process regression was used to ensure estimates were complete and comparable across time and to generate uncertainty.

**Findings:**

In 2016, US$4·3 billion (95% uncertainty interval [UI] 4·2–4·4) was spent on malaria worldwide, an 8·5% (95% UI 8·1–8·9) per year increase over spending in 2000. Since 2000, OOP spending increased 3·8% (3·3–4·2) per year, amounting to $556 million (487–634) or 13·0% (11·6–14·5) of all malaria spending in 2016. Governments spent $1·2 billion (1·1–1·3) or 28·2% (27·1–29·3) of all malaria spending in 2016, increasing 4·0% annually since 2000. The source of malaria spending varied depending on whether countries were in the malaria control or elimination stage.

**Interpretation:**

Tracking global malaria spending provides insight into how far the world is from reaching the malaria funding target of $6·6 billion annually by 2020. Because most countries with a high burden of malaria are low income or lower-middle income, mobilising additional government resources for malaria might be challenging.

**Funding:**

The Bill & Melinda Gates Foundation.

## Introduction

Between 2000 and 2017, tremendous progress was made in the fight against malaria, although the disease burden remains high in many countries.^1^ Globally, malaria incidence fell by nearly 1% annually or 1·9 million cases per year and malaria death rates dropped 3·1% each year, averting an additional 14 000 deaths annually on average. Relative to 2000, 24·2 million fewer malaria cases and 226 000 fewer deaths occurred in 2016.^2^, ^3^, ^4^ These declines were underpinned by a 30·2% increase in development assistance for malaria between 2000 and 2010.^5^ More than US$10·3 billion in development assistance for health (DAH) was disbursed to control and eliminate malaria between 2000 and 2010.

Despite this progress, a large malaria health burden persists in many low-income and lower-middle-income countries and particularly in sub-Saharan Africa.^6^ In 2017, 208·8 million cases of malaria occurred globally and 620 000 people died, including 328 000 children under the age of 5 years in sub-Saharan Africa.^3^, ^4^, ^7^, ^8^ Evidence suggests progress in the fight against malaria has stalled in some settings.^9^ Moreover, between 2010 and 2018, development assistance for malaria decreased 1·9% annually on average.

Sustaining achievements in malaria control and making progress towards global malaria elimination goals requires funding above present levels of DAH. The Global Technical Strategy for Malaria, 2016–30, aims to reduce case incidence and mortality by 40% by 2020, eliminate malaria from at least ten countries, and prevent re-introduction of malaria in all malaria-free settings.^10^ Achieving these aims requires an estimated $6·6 billion in malaria investments annually by 2020.^11^, ^12^

With little growth in future DAH expected, it is crucial to better understand present health spending on malaria, including how much is financed by governments, households, and prepaid private sources in malaria-endemic countries.^5^ To date, no comprehensive and comparable estimates of global spending on malaria exist. Country-specific information has increased, with malaria spending estimates for 149 years of data for numerous countries published in National Health Account (NHA) sub-accounts and System of National Health Accounts 2011 reports.^13^ However, just 55 of these country-years accounted for malaria out-of-pocket (OOP) payments, which comprise spending by households at the point of care and other household spending to prevent and treat malaria. Estimates of government health spending on malaria have been published for all malaria-endemic countries and 35 malaria elimination countries.^11^, ^14^, ^15^, ^16^ However, these estimates omitted government spending on inpatient and outpatient care for malaria. Overall, incomplete estimates of government malaria spending and few credible studies on malaria OOP expenditure limit knowledge about the full malaria spending envelope—information which is important for tracking progress towards malaria reduction and elimination targets.

**Research in context**

**Evidence before this study**

Development assistance for malaria has been estimated annually by the Institute for Health Metrics and Evaluation since 2010. Since 2009, national malaria control programmes have reported their spending to WHO, which published these values in World Malaria Reports, although these country-level estimates excluded spending on malaria patient care. Similarly, previous research that estimated government spending on malaria globally for 35 malaria elimination countries excluded patient care. To date, no comprehensive global estimates of total and out-of-pocket (OOP) spending on malaria have been published. National Health Accounts, some of which follow new System of Health Accounts 2011 guidelines for disaggregating disease-specific spending, captured 55 country-years of OOP spending. WHO estimated global OOP spending on malaria in 2015 but did not include OOP spending on treatment or antimalarial medicine other than artemisinin-based combination therapy. A PubMed search of “malaria out-of-pocket spending”, “malaria financing”, and “malaria direct costs” for research published between 2000 and 2018 yielded 31 individual studies on OOP spending in, at most, three countries. Estimates of OOP from these studies were not equivalent to each other because the estimates were based on a diverse set of methods and did not have nationally representative samples in many cases. In general, there is a major gap in what is known about total, government, and OOP spending on malaria.

**Added value of this study**

This study generated, for the first time, a complete set of total malaria spending estimates, spanning 2000 to 2016, for 106 malaria-endemic countries and countries that eliminated malaria after 2000. Government spending that included malaria patient care and OOP malaria expenditure were also estimated by country for the first time. More than 36 038 datapoints on malaria spending, volume of care-seeking, drug prices, and cost of malaria patient care were collected, collated, standardised, and combined to produce these estimates. By use of spatiotemporal Gaussian process regression, malaria spending estimates were produced that had uncertainty quantified and were comprehensive and equivalent across countries and time.

**Implications of all the available evidence**

In 2016, US$4·3 billion (95% uncertainty interval [UI] 4·2–4·4) was spent on malaria worldwide. This falls short of the estimated $6·6 billion per year required by 2020 to meet global malaria elimination goals. Since 2000, total OOP has increased by 3·8% (95% UI 3·3–4·2) per year and government spending on malaria has increased by 4·0% (95% UI 3·8–4·2) per year, although, because of the surge in development assistance for malaria since 2000, both sources of financing have declined as a share of total malaria spending. In malaria control countries, governments contributed a smaller share of malaria spending (28·3% [26·7–29·9] or $739 million [688–790]) than in malaria elimination countries (47·9% [45·4–50·4] or $409 million [374–447]). In control countries, where 86·0% of all incident cases occurred in 2016, OOP comprised a large share of spending at 19·0% (16·9–21·4) or $497 million (430–574) and development assistance for malaria comprised 50·0% (48·3–51·7) or $1·3 billion. The burden of financing placed on households could deter access to needed treatment, potentially stifling broader efforts to reach global malaria elimination goals, including efforts to reduce onward transmission and malaria mortality. Furthermore, because many malaria control countries are low income, raising more government resources to reduce OOP and reach funding targets might be challenging. Efforts are needed to ensure sufficient resources for reducing malaria incidence and for moving closer to malaria eradication.

The objective of our study was to estimate malaria spending from 2000 to 2016. We focused on the 106 countries that were malaria-endemic during this period, including countries that became malaria-free any time after 2000. First, we estimated government malaria spending sourced domestically, including spending on malaria inpatient and outpatient care. Second, we estimated private spending on malaria, both household OOP spending and prepaid private spending. Finally, we combined government, OOP, and prepaid private spending estimates with estimates of development assistance for malaria published by the Institute for Health Metrics and Evaluation to generate total spending on malaria in all 106 countries. Overall, our analysis aims to provide information crucial to assessing all resources used to prevent and treat malaria. This evidence is needed to establish whether more funding needs to be mobilised to reach global malaria goals.

## Methods

### Study design

Domestic malaria spending was estimated for three financing sources: government, OOP, and prepaid private spending. A different strategy was developed for each domestic financing source. Development assistance for malaria, or the financial and in-kind resources provided by development agencies to low-income and middle-income countries for the primary purpose of preventing or treating malaria, was drawn from public databases published by the Institute for Health Metrics and Evaluation.^5^ All spending estimates are reported in 2018 US$ (see appendix section S1 for further information on currency conversions).

### Government health spending

We used three sources of data that provided aggregated information on government malaria spending to estimate government spending on malaria. First, we extracted government malaria spending estimates reported by countries in 134 concept notes and 224 proposals submitted to the Global Fund.^17^ Second, we used the government spending datapoints reported in 86 NHAs, including malaria sub-accounts and reports based on the System of Health Accounts 2011 guidelines.^18^ Finally, we extracted 785 government malaria spending estimates reported in WHO's World Malaria Reports, published between 2009 and 2018.^12^

The government malaria spending estimates reported to World Malaria Reports and the Global Fund did not include spending by governments on inpatient and outpatient care for malaria.^12^ To ensure our estimates included all government spending on malaria, as in the NHAs, we estimated spending on malaria patient care—namely, the costs of staff and facilities (ie, spending on malaria excluding drugs and diagnostics, which would be purchased by national malaria control programmes) used for malaria treatment that occurred in government-run health facilities.

We estimated government spending on inpatient and outpatient care separately. The estimation strategy involved combining estimates of volume and price of malaria treatment as shown in equation 1:

$$\begin{array}{l} \text{gov spending patient}{\text{care}}_{\text{mal}}\\ =\left(\text{gov cost per}{\text{admission}}_{\text{mal}}\times \text{public}{\text{admissions}}_{\text{mal}}\right)\\ +\left(\text{gov cost per outpatient}{\text{visit}}_{\text{mal}}\times \text{public}{\text{outpatient visits}}_{\text{mal}}\right) \end{array}$$

For volume of care, the number of people who sought care for treatment in government facilities was based on published^19^ and updated (personal correspondence with Katherine E Battle, University of Oxford) estimates by Battle and colleagues of public treatment seeking and estimates of treatment-seeking for fever among children aged under 5 years, based on household surveys (3536 datapoints for the 106 countries in our study). For volume of inpatient visits, we used counts of inpatient cases (574) reported in World Malaria Reports and estimated a full time series of inpatient admissions by means of spatiotemporal Gaussian process regression (ST-GPR).^20^ ST-GPR was developed as part of the Global Burden of Disease study to identify patterns across time and space. To calculate public inpatient stays, we multiplied inpatient cases by the ratio of public treatment-seeking over all treatment-seeking from Battle and colleagues.^19^ To estimate public outpatient visits, we subtracted public admissions from the public treatment-seeking estimates from Battle and colleagues.

For price, we used 3604 datapoints representing average inpatient and outpatient unit costs for all health conditions from Moses and colleagues.^21^ We scaled these unit costs to the average cost of a malaria inpatient and outpatient visit (excluding drug and diagnostics spending), on the basis of existing literature (appendix section S2). Finally, the volume and unit cost estimates were multiplied and summed to estimate government spending on malaria patient care.

We added the estimates of government spending on malaria patient care to the government spending reported in the World Malaria Reports and Global Fund concept notes and proposals. These sums were appended to government spending from the NHAs, which include patient care and national malaria control programme spending. We then applied ST-GPR to these data, with covariates selected on the basis of out-of-sample root-mean-square error, 15 outlier points omitted on the basis of Cooke's distance, and uncertainty generated through the Gaussian process. We ran separate ST-GPR models for sub-Saharan Africa and countries outside sub-Saharan Africa because of the distinct patterns of malaria incidence and malaria control efforts prevalent in the different regions. Extensive details on each step in the analysis are included in the appendix (section S2).

### Out-of-pocket spending

OOP malaria spending was reported in 55 NHA country-years. To augment these data, we estimated OOP spending on the basis of volumes and OOP costs of treatment. We assumed OOP spending was primarily on treatment because of the large investments governments and development assistance partners have made in prevention efforts, including the provision of commodities such as insecticide-treated nets free of charge.^5^, ^12^ We modelled OOP spending on inpatient stays, outpatient visits, and drugs separately, including artemisinin-based combination therapy (ACT), with estimates of the volume and price of each type of treatment multiplied and summed to estimate OOP spending. These were combined by means of equation 2:

$$\begin{array}{l} {\text{OOP}}_{\text{mal}}=\left(\text{OOP cost per}{\text{admission}}_{\text{mal}}\times {\text{admissions}}_{\text{mal}}\right)\\ +\left(\text{OOP cost per outpatient}{\text{visit}}_{\text{mal}}\times \text{outpatient}{\text{visits}}_{\text{mal}}\right)\\ +\left(\text{OOP ACT cost}\times \text{ACT courses}\right)\\ +\left(\text{OOP other antimalarial cost}\times \text{other antimalarial courses}\right) \end{array}$$

For the volume of inpatient care, we used the full time series of estimated inpatient admissions described previously. Average inpatient unit costs for all health conditions were drawn from Moses and colleagues.^21^ To estimate the share of inpatient unit costs sourced OOP, we used 471 NHA datapoints capturing OOP inpatient spending as a share of total inpatient expenditure and estimated a full time series with ST-GPR. The estimated fraction was applied to the inpatient unit costs to generate OOP inpatient unit costs. These OOP costs were scaled to malaria OOP unit costs from the literature on malaria-specific OOP inpatient unit costs (appendix section S3).

OOP spending on outpatient visits was estimated in a similar manner. For volume, we subtracted malaria inpatient admissions from the malaria treatment-seeking estimates drawn from Battle and colleagues.^19^ To estimate price, average outpatient unit costs for all health conditions were drawn from Moses and colleagues.^21^ These were multiplied by the share of outpatient spending sourced OOP, estimated by applying ST-GPR to 471 NHA datapoints. These OOP unit costs were scaled to malaria OOP outpatient unit costs, excluding the costs of drugs, on the basis of existing literature (appendix section S3).

Finally, we estimated OOP spending on antimalarial medicine. For the volume of malaria treatment courses, we subtracted malaria inpatient stays from malaria treatment-seeking estimates from Battle and colleagues.^19^ Because the costs differ, we estimated volumes separately for ACT and other malaria drugs.^19^ Estimates of ACT treated cases were sourced from the Malaria Atlas Project and the Global Burden of Disease study.^22^, ^23^ We subtracted ACT treated cases from estimates of treatment-seeking to generate the number of other malaria treatment courses taken. For the average drug price, we sourced data from ACT Watch (145 datapoints), the Health Access Initiative (260 datapoints), and an Affordable Medicines Facility–malaria (AMF-m) report (127 datapoints).^24^, ^25^ To ensure our estimates were robust to outliers without eliminating datapoints, we used robust regression to model ACT prices. Because the prices of other malaria drugs have not changed substantially over time, we only considered country and regional variation in the price of other antimalarials, which was modelled with a linear mixed effects model.^26^

To estimate total malaria OOP, we first summed the estimates of OOP spending on inpatient stays, outpatient visits, and malaria treatment courses, as shown in equation 2. We appended these data to the NHA OOP spending estimates and eliminated 46 outlier points with Cooke's distance. Because of the distinct malaria incidence and intervention patterns in different regions, we ran separate ST-GPR models for sub-Saharan Africa and other regions, with uncertainty generated through the Gaussian process. More details on the estimation procedure for each component, including the peer-reviewed studies used for unit cost adjustments, are in the appendix (section S3).

### Prepaid private spending

We found 31 country-years of data on prepaid private spending reported in available NHAs. In these data, prepaid private constituted 1·5% (IQR 0·8–6·8) of total malaria spending at the median. Despite this small amount, generating a comprehensive total malaria spending estimate requires an estimate of prepaid private malaria spending for each country-year. To generate these estimates, we first estimated the ratio of prepaid health spending relative to all other health spending for each country. These total health spending estimates were drawn from the Institute for Health Metrics and Evaluation's Health financing database.^5^ Then, using the 31 country-years of commensurate malaria spending data from the NHAs, we calculated the same ratio, although for malaria spending. We then compared these malaria spending ratios with the health spending ratios by use of equation 3:

$$\begin{array}{l} \frac{\text{prepaid}{\text{private}}_{\text{mal}}}{{\text{gov}}_{\text{mal}}+{\text{OOP}}_{\text{mal}}+{\text{DAH}}_{\text{mal}}}\\ =\rho \frac{\text{prepaid private health}}{\text{gov health}+\text{OOP health}+\text{DAH}} \end{array}$$

The median of the 31 ρ (one for each country-year of NHA data) was 0·36. To estimate prepaid private malaria spending for each country-year, we adjusted the observed ratio of prepaid to government, OOP, and donor health spending by multiplying it by 0·36 (see appendix section S4 for further information on estimating prepaid private spending).

### Aggregation, currency conversions, and sensitivity analyses

We aggregated our health spending estimates by categorising countries by income group, geographical region, and malaria disease burden. We used 2018 World Bank income groups and Global Burden of Disease regional groupings.^22^, ^27^ Estimates were calculated by aggregating spending by each group and dividing by population or malaria incident cases across the entire group or region. These estimates represent the grouping as a whole rather than an average of the countries. To examine malaria spending, countries were grouped into three categories as determined by the Malaria Elimination Initiative at the University of California, San Francisco: malaria-free, malaria elimination, and malaria control countries (countries listed in appendix section S5). Finally, we present data availability across regions and elimination status for all the data inputs to our study (appendix section S6).

We assessed the sensitivity of our results to inputs with sparse data. We estimated OOP and government spending on malaria on the basis of the 25th and 75th percentiles of inpatient and outpatient malaria unit costs. OOP spending is also quantified with the 25th and 75th percentiles of OOP drug unit costs. Finally, we used the 25th and 75th percentiles of ρ to examine variation in prepaid private malaria spending. These results can be found in appendix section S7.

The analysis was done with R (version 3.4.0) and Stata (version 13).

### Role of the funding source

The funder of this study had no role in study design, data collection, data analysis, data interpretation, or writing of the manuscript. All authors had full access to all the data in the study, and JLD and CJLM had final responsibility for the decision to submit for publication.

## Results

**Figure 1:**
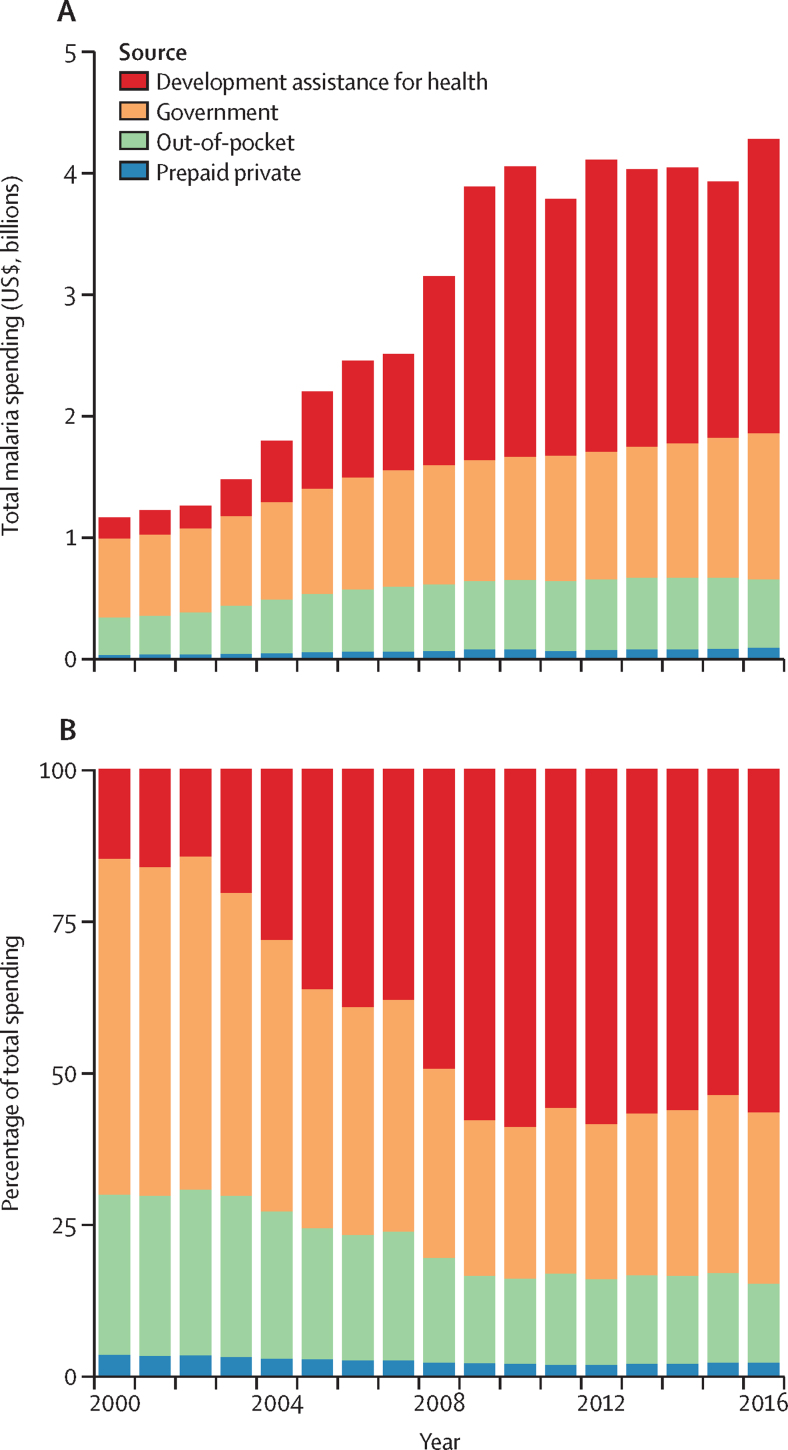
Malaria spending by source, 2000–16 Total malaria spending over time by source (A) and scaled to represent 100% of all malaria spending (B). Estimates shown in 2018 US$.

Figure 1 represents total spending on malaria in the 106 countries in our study between 2000 and 2016, broken down by the source of financing. Total spending on malaria in these countries increased at an annualised rate of 8·5% (95% uncertainty interval [UI] 8·1–8·9), from $1·2 billion (95% UI 1·1–1·2) in 2000 to $4·3 billion (4·2–4·4) in 2016. Over this period, the sources of funding for malaria shifted substantially. In 2000, malaria OOP spending amounted to $307 million (270–348) or 26·4% (23·9–29·2) of all malaria spending, and government expenditure constituted $643 million (602–687) or 55·3% (52·9–57·6). By 2016, total government spending on malaria amounted to $1·2 billion (1·1–1·3) or 28·2% (27·1–29·3) of all malaria spending. By 2016, OOP malaria spending as a share of total malaria spending declined to 13·0% (11·6–14·5), although total OOP malaria spending in 2016 ($556 million, 95% UI 487–634) was higher than in 2000. Finally, development assistance for malaria comprised 56·5% or $2·4 billion and prepaid private spending constituted 2·3% (2·1–2·6) or $99 million (88–112) in 2016.

**Figure 2:**
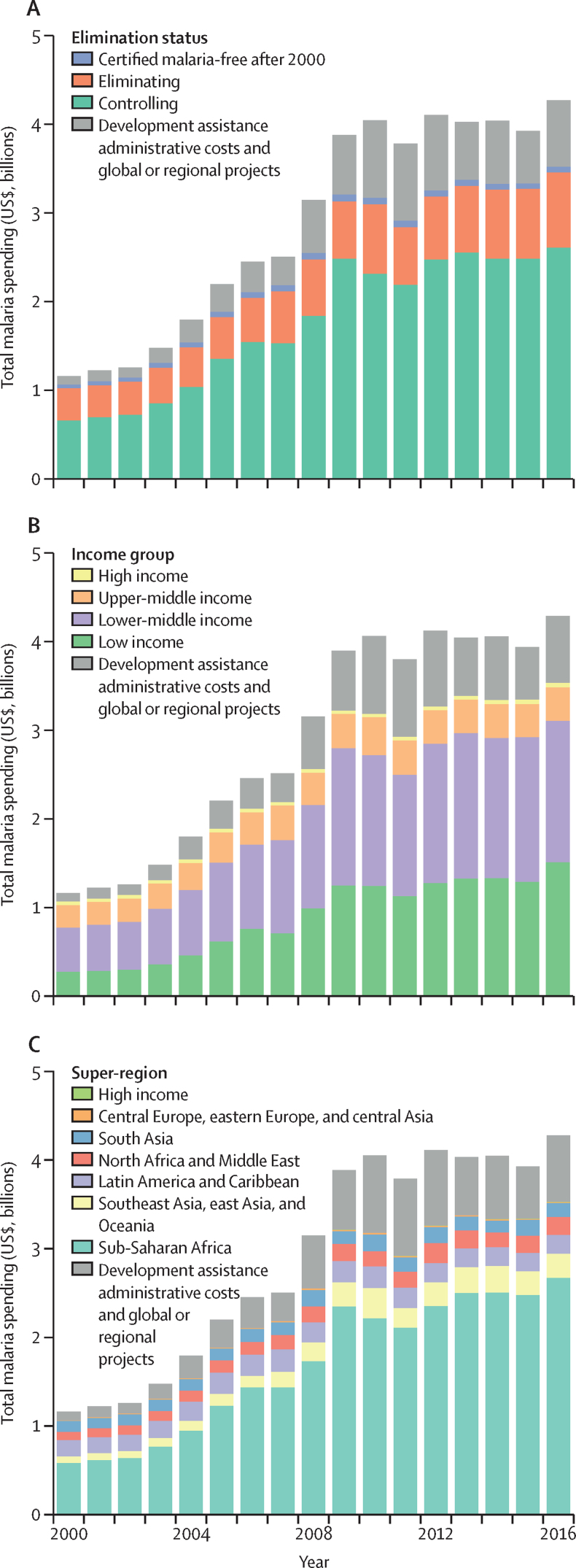
Malaria spending by country characteristics, 2000–16 Total malaria spending by 2016 malaria elimination status (A), 2018 World Bank income group (B), and 2017 Global Burden of Disease region (C). Income group specifies where the resources were spent, not the source of the funds; very few resources were spent in the high-income super-region. Estimates are shown in 2018 US$.

Total malaria spending varied depending on region, malaria elimination status, and income group (figure 2). Over 2000–16, the bulk of malaria spending occurred in the 47 control countries: 72·8% (95% UI 71·8–73·9) or $29·9 billion (95% UI 28·9–31·0), with $2·6 billion (2·5–2·7) spent in 2016. The 41 eliminating countries were where 24·6% (23·6–25·6) or $10·1 billion (9·7–10·5) of malaria spending occurred over 2000–16, a total of $854 million (812–900) in 2016. Across 2018 World Bank income groups, highest malaria spending over 2000–16 was in lower-middle-income countries (47·1%, 45·6–48·5; $19·3 billion, 18·4–20·4), followed by low-income countries (36·9%, 35·7–38·0; $15·1 billion, 14·8–15·5), excluding DAH costs. Globally, 86·2% of all malaria cases occurred in sub-Saharan Africa in 2016. Malaria spending was also concentrated in the region: 69·7% (68·4–70·9) or $28·6 billion (27·7–29·7) of all malaria spending took place in sub-Saharan Africa over 2000–16, with a total of $2·7 billion (2·6–2·8 billion) in 2016. A portion of global spending on malaria, 11·5% (11·3–11·8) or $493 million, is disbursed as development assistance administrative costs and global or regional projects, which represents spending on global and regional convenings, guideline-setting, coordination, and other global or regional efforts tackling malaria.

Trends were also distinct by country groupings. Since 2000, governments have increased their investments annually in both control (4·7%, 95% UI 4·3 to 5·1) and elimination countries (3·2%, 2·8 to 3·6). Between 2000 and 2016, OOP spending grew 4·5% (4·1 to 4·9) per year in control countries and decreased −0·3% (−0·7 to 1·1) per year in eliminating countries. Examining trends in malaria spending by income, annual growth rates were most substantial in low-income (11·1%, 10·7–11·6) and lower-middle income countries (7·5%, 7·1–8·0). Malaria spending increased 2·5% (2·0–2·9) per year on average in upper-middle-income countries.

**Figure 3:**
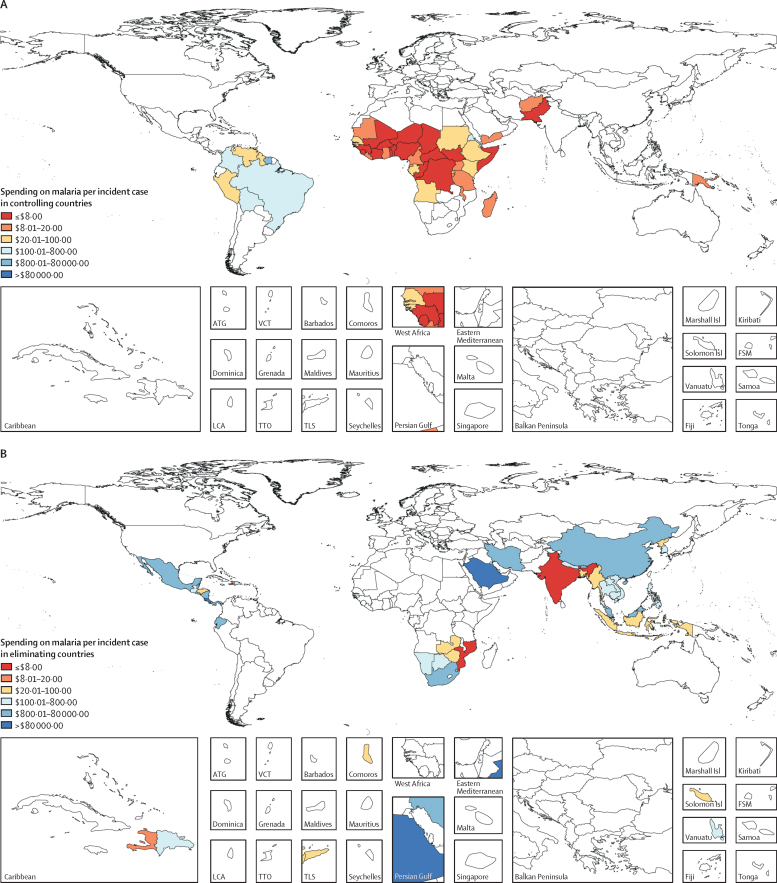

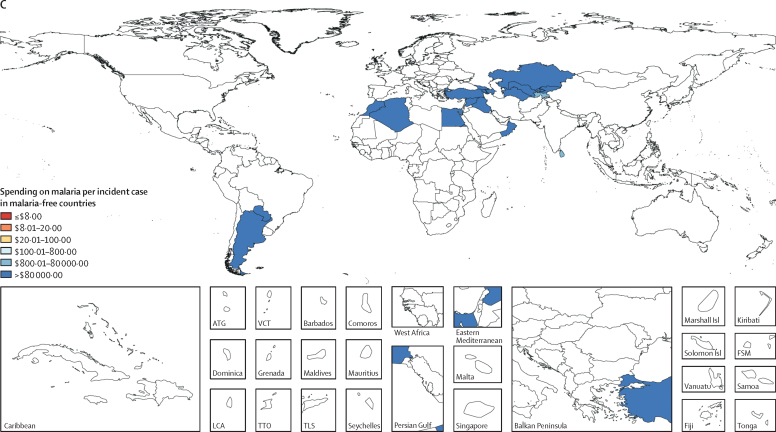
Malaria spending per malaria incident case in controlling (A), eliminating (B), and malaria-free (C) countries, 2016 Estimates are shown in 2018 US$. ATG=Antigua and Barbuda. FSM=Federated States of Micronesia. LCA=Saint Lucia. Marshall Isl=Marshall Islands. Solomon Isl=Solomon Islands. TLS=Timor-Leste. TTO=Trinidad and Tobago. VCT=Saint Vincent and the Grenadines.

Substantial variation in 2016 total spending on malaria per incident case is shown in figure 3. Malaria spending per incident case was $14·07 (95% UI 13·61–14·56) on average in malaria control countries, with an interquartile range of $10·45–52·77 per incident case. In malaria elimination countries, where incidence is lower and spending focuses on surveillance and prevention activities predominately, spending per incident case was higher on average, at $28·12 (26·76–29·66).

Shown in the table is the share of malaria spending by source. In malaria control countries, $739 million (95% UI 688–790) was sourced from governments in 2016. As a share of spending, governments in control countries contributed 53·7% (95% UI 50·2–57·0) in 2000, substantially more than government contributions in 2016 (28·3%, 26·7–29·9). Government spending on malaria constituted much more of the malaria spending in elimination countries (47·9%, 45·4–50·4; $409 million, 374–447). Households spent 19·0% (16·9–21·4) or $497 million (430–574) OOP in malaria control countries in 2016, a drop from the proportion contributed in 2000 (36·8%, 33·2–40·6). The OOP share is lower in malaria elimination countries (7·0%, 5·2–9·6; $60 million, 43–84), where less spending on malaria treatment takes place. More malaria development assistance was disbursed in control countries (50·0% or $1·3 billion) than in malaria elimination countries (42·0% or $358 million) in 2016. Some malaria control countries, such as Madagascar and Afghanistan, are substantially more dependent on development assistance, with more than 85% of all malaria spending sourced externally in 2016.

**Table:** Total malaria spending and spending by source, 2016

		**Total spending on malaria (US$, millions)**	**Percentage of malaria spending that is development assistance**	**Percentage of malaria spending that is government spending**	**Percentage of malaria spending that is out-of-pocket**	**Percentage of development assistance for health that is for malaria**	**Percentage of government health spending that is for malaria**
106 malaria-endemic countries		4277·7 (4177·8–4383·0)	56·5% (55·2–57·9)	28·2% (27·1–29·3)	13·0% (11·6–14·5)	10·0% (10·0–10·0)	0·2% (0·1–0·2)
Malaria elimination stage							
	Controlling	2612·6 (2526·8–2704·0)	50·0% (48·3–51·7)	28·3% (26·7–29·9)	19·0% (16·9–21·4)	13·2% (13·2–13·2)	0·6% (0·5–0·7)
	Eliminating	853·7 (812·2–900·3)	42·0% (39·8–44·1)	47·9% (45·4–50·4)	7·0% (5·2–9·6)	6·1% (6·1–6·1)	0·1% (0·1–0·1)
	Malaria-free	61·7 (54·5–70·1)	5·2% (4·5–5·8)	93·0% (92·1–93·8)	0·0% (0·0–0·0)	0·3% (0·3–0·3)	0·1% (0·1–0·1)
World Bank income group							
	High income	42·5 (31·2–58·9)	0·0% (0·0–0·0)	93·8% (92·9–94·6)	0·1% (0·1–0·2)	..	0·0% (0·0–0·1)
	Upper-middle income	378·8 (344·1–417·6)	6·3% (5·7–6·9)	85·4% (84·4–86·3)	2·1% (1·7–2·5)	1·2% (1·2–1·2)	0·1% (0·0–0·1)
	Lower-middle income	1606·9 (1523·8–1694·0)	43·1% (40·8–45·4)	32·4% (30·1–35·0)	21·9% (18·9–25·5)	9·1% (9·1–9·1)	0·7% (0·6–1·0)
	Low income	1499·9 (1464·4–1541·9)	63·6% (61·8–65·1)	21·4% (20·1–22·8)	13·0% (11·6–14·6)	13·3% (13·3–13·3)	4·8% (3·6–6·2)
Global Burden of Disease super-region							
	Central Europe, eastern Europe, and central Asia	11·4 (9·7–13·2)	3·4% (2·9–3·9)	95·3 (94·5–96·0)	0·0% (0·0–0·0)	0·1% (0·1–0·1)	0·2% (0·1–0·2)
	High income	2·2 (1·7–2·8)	0·2% (0·2–0·2)	94·2% (92·5–95·4)	2·6% (1·5–4·3)	0·0% (0·0–0·0)	0·0% (0·0–0·0)
	Latin America and Caribbean	210·3 (181·5–244·6)	10·8% (9·3–12·5)	82·1% (80·4–83·7)	0·9% (0·6–1·2)	2·2% (2·2–2·2)	0·1% (0·1–0·1)
	North Africa and Middle East	206·0 (188·2–226·2)	34·5% (31·3–37·6)	55·3% (51·3–59·4)	7·9% (5·3–11·1)	8·9% (8·9–8·9)	0·1% (0·1–0·1)
	South Asia	153·4 (126·2–186·2)	18·5% (15·1–22·2)	53·1% (42·9–62·3)	25·3% (16·8–36·3)	1·5% (1·5–1·5)	0·3% (0·2–0·5)
	Southeast Asia, east Asia, and Oceania	269·8 (251·8–290·6)	47·0% (43·6–50·3)	44·6% (40·9–48·2)	5·7% (4·2–7·7)	7·9% (7·9–7·9)	0·0% (0·0–0·0)
	Sub-Saharan Africa	2675·0 (2592·3–2766·7)	53·1% (51·3–54·8)	26·3% (24·8–27·8)	18·1% (16·0–20·4)	13·1% (13·1–13·1)	2·5% (2·0–3·0)
Country							
	Afghanistan (C)	8·6 (8·3–9·1)	85·1% (80·2–88·7)	6·4% (4·5–8·5)	8·4% (5·0–13·5)	4·1% (4·1–4·1)	0·5% (0·3–0·8)
	Algeria (F)	7·2 (5·0–10·0)	0·0% (0·0–0·0)	99·5% (99·0–99·7)	0·0% (0·0–0·0)	0·0% (0·0–0·0)	0·1% (0·1–0·1)
	Angola (C)	119·9 (98·5–145·9)	24·7% (20·1–29·8)	59·4% (52·0–66·4)	9·3% (6·1–13·6)	26·1% (26·1–26·1)	4·8% (3·3–7·0)
	Argentina (F)	1·2 (0·8–1·6)	0·4% (0·3–0·5)	96·5% (95·5–97·2)	0·0% (0·0–0·0)	0·0% (0·0–0·0)	0·0% (0·0–0·0)
	Armenia (F)	0·2 (0·1–0·3)	0·0% (0·0–0·0)	99·6% (99·2–99·8)	0·0% (0·0–0·0)	0·0% (0·0–0·0)	0·1% (0·1–0·2)
	Azerbaijan (F)	2·2 (1·6–3·0)	3·1% (2·2–4·2)	96·7% (95·5–97·5)	0·0% (0·0–0·0)	0·7% (0·7–0·7)	0·4% (0·2–0·5)
	Bangladesh (E)	9·8 (9·1–10·6)	77·0% (71·2–82·4)	21·6% (16·4–27·6)	0·4% (0·2–0·7)	1·9% (1·9–1·9)	0·2% (0·1–0·3)
	Belize (E)	0·3 (0·2–0·4)	0·4% (0·3–0·5)	97·1% (95·2–98·4)	0·0% (0·0–0·0)	0·0% (0·0–0·0)	0·4% (0·3–0·6)
	Benin (C)	30·5 (27·6–33·6)	55·0% (49·8–60·6)	26·0 (20·3–31·2)	16·8 (11·3–23·5)	17·0 (17·0–17·0)	10·1 (6·8–14·9)
	Bhutan (E)	0·7 (0·7–0·8)	67·0% (59·3–74·1)	32·6% (25·5–40·4)	0·0% (0·0–0·0)	12·3% (12·3–12·3)	0·5% (0·3–0·7)
	Bolivia (C)	6·7 (6·2–7·4)	70·8% (64·1–77·2)	27·8% (21·2–34·6)	0·2% (0·1–0·3)	11·0% (11·0–11·0)	0·1% (0·1–0·2)
	Botswana (E)	2·0 (1·5–2·7)	7·6% (5·6–10·0)	77·7% (73·7–81·4)	0·4% (0·2–0·6)	0·2% (0·2–0·2)	0·3% (0·2–0·4)
	Brazil (C)	83·6 (60·0–117·1)	0·3% (0·2–0·4)	89·2% (87·4–90·9)	1·1% (0·6–1·9)	0·1% (0·1–0·1)	0·1% (0·1–0·1)
	Burkina Faso (C)	107·9 (95·3–122·9)	44·3% (38·7–50·0)	30·9% (24·8–37·3)	22·4% (16·1–30·4)	31·0% (31·0–31·0)	13·7% (9·3–19·6)
	Burundi (C)	33·8 (30·9–37·0)	59·4% (54·2–64·7)	30·0% (24·4–35·8)	10·0% (6·7–14·3)	13·2% (13·2–13·2)	12·2% (8·2–17·7)
	Cambodia (E)	16·1 (15·2–17·1)	78·5% (73·7–83·0)	13·8% (10·0–18·0)	7·5% (4·5–12·0)	8·2% (8·2–8·2)	0·8% (0·5–1·2)
	Cameroon (C)	77·6 (62·2–95·4)	15·3% (12·3–18·9)	32·2% (24·2–41·0)	51·6% (41·2–61·9)	9·3% (9·3–9·3)	12·1% (8·1–17·5)
	Cape Verde (E)	0·8 (0·6–1·0)	4·2% (3·1–5·6)	94·8% (93·2–96·1)	0·1% (0·1–0·2)	0·7% (0·7–0·7)	1·3% (0·9–1·8)
	Central African Republic (C)	4·3 (3·9–4·7)	63·5% (57·7–69·4)	16·7% (12·7–21·0)	19·4% (13·6–26·3)	5·2% (5·2–5·2)	5·0% (3·3–7·3)
	Chad (C)	42·6 (40·9–44·8)	81·3% (77·3–84·8)	8·1% (6·0–10·7)	8·7% (5·8–12·6)	45·5% (45·5–45·5)	3·1% (2·0–4·6)
	China (E)	18·7 (13·0–25·5)	3·1% (2·2–4·4)	94·6% (92·6–96·1)	0·1% (0·1–0·2)	0·3% (0·3–0·3)	0·0% (0·0–0·0)
	Colombia (C)	17·9 (13·1–24·4)	5·8% (4·1–7·7)	87·4% (84·0–90·3)	1·2% (0·6–2·2)	7·3% (7·3–7·3)	0·1% (0·1–0·2)
	Comoros (E)	3·6 (3·5–3·7)	85·1% (82·1–87·8)	9·6% (7·4–12·2)	4·9% (3·4–7·0)	27·9% (27·9–27·9)	4·4% (2·9–6·3)
	Congo (C)	11·3 (8·7–14·2)	0·6% (0·4–0·7)	70·4% (59·8–79·3)	27·5% (18·4–38·1)	0·4% (0·4–0·4)	4·6% (3·1–7·0)
	Costa Rica (E)	6·7 (4·7–9·2)	0·0% (0·0–0·0)	99·0% (98·3–99·5)	0·0% (0·0–0·0)	0·0% (0·0–0·0)	0·2% (0·1–0·3)
	Côte d'Ivoire (C)	91·7 (83·9–101·8)	61·2% (55·1–66·8)	23·2% (18·1–28·6)	7·9% (5·2–11·1)	22·1% (22·1–22·1)	5·2% (3·4–7·9)
	Democratic Republic of the Congo (C)	189·2 (174·7–208·9)	66·3% (59·9–71·7)	9·5% (7·1–12·3)	21·2% (15·1–28·7)	22·7% (22·7–22·7)	8·15 (5·3–12·2)
	Djibouti (C)	7·0 (6·5–7·6)	71·4% (65·6–77·1)	28·0% (22·4–33·9)	0·3% (0·2–0·4)	34·2% (34·2–34·2)	5·9% (3·9–8·4)
	Dominican Republic (E)	3·1 (2·2–4·3)	0·3% (0·2–0·5)	96·0% (93·8–97·5)	0·2% (0·1–0·3)	0·0% (0·0–0·0)	0·1% (0·1–0·2)
	Ecuador (E)	5·9 (4·2–8·2)	2·9% (2·0–3·9)	94·2% (92·3–95·7)	0·2% (0·1–0·3)	1·0% (1·0–1·0)	0·1% (0·1–0·2)
	Egypt (F)	3·4 (2·5–4·6)	7·8% (5·6–10·6)	89·2% (85·8–92·0)	0·0% (0·0–0·0)	0·5% (0·5–0·5)	0·1% (0·1–0·1)
	El Salvador (E)	3·2 (2·2–4·3)	0·1% (0·0–0·1)	97·6% (95·8–98·7)	0·0% (0·0–0·0)	0·0% (0·0–0·0)	0·2% (0·2–0·4)
	Equatorial Guinea (C)	9·3 (7·5–11·3)	0·6% (0·5–0·7)	62·9% (54·1–71·9)	35·0% (26·2–43·6)	0·8% (0·8–0·8)	10·6% (7·3–14·6)
	Eritrea (C)	8·8 (8·4–9·4)	79·3% (74·3–83·4)	18·4% (14·2–23·4)	1·7% (1·1–2·4)	29·9% (29·9–29·9)	5·1% (3·3–7·5)
	eSwatini (E)	2·9 (2·5–3·3)	51·1% (43·9–58·1)	46·0% (39·1–53·4)	0·1% (0·1–0·2)	1·5% (1·5–1·5)	0·5% (0·4–0·7)
	Ethiopia (C)	81·2 (72·9–92·1)	57·0% (50·1–63·3)	32·7% (26·2–39·3)	3·5% (2·2–5·2)	5·6% (5·6–5·6)	3·8% (2·5–5·8)
	Gabon (C)	20·9 (16·3–26·7)	1·4% (1·1–1·8)	83·1% (78·1–87·6)	10·8% (7·0–15·6)	5·1% (5·1–5·1)	5·7% (4·0–7·9)
	The Gambia (C)	4·0 (3·9–4·2)	81·4% (77·4–84·8)	13·3% (10·4–16·8)	2·0% (1·3–2·8)	9·9% (9·9–9·9)	5·8% (3·9–8·6)
	Georgia (F)	2·5 (1·8–3·4)	1·5% (1·1–2·1)	96·4% (94·8–97·6)	0·0% (0·0–0·0)	0·2% (0·2–0·2)	0·6% (0·4–0·8)
	Ghana (C)	172·0 (149·3–197·7)	44·3% (38·3–50·8)	28·2% (21·6–35·2)	24·8% (17·6–33·2)	26·5% (26·5–26·5)	5·9% (3·8–8·4)
	Guatemala (E)	4·8 (3·9–5·9)	37·1% (29·6–45·2)	60·0% (52·0–67·4)	0·2% (0·1–0·3)	3·3% (3·3–3·3)	0·2% (0·1–0·3)
	Guinea (C)	33·0 (30·3–36·3)	60·8% (55·2–66·1)	17·3% (13·4–21·6)	18·1% (12·2–24·3)	13·8% (13·8–13·8)	9·4% (6·1–13·4)
	Guinea-Bissau (C)	11·3 (10·9–11·8)	83·6% (80·1–86·8)	13·4% (10·4–16·9)	3·0% (2·0–4·4)	31·9% (31·9–31·9)	4·8% (3·2–6·8)
	Guyana (C)	1·6 (1·2–2·1)	8·4% (6·2–11·2)	89·3% (85·8–92·2)	2·2% (1·2–3·8)	1·8% (1·8–1·8)	1·6% (1·1–2·4)
	Haiti (E)	5·8 (5·6–6·0)	86·8% (83·2–89·9)	10·0% (7·1–13·3)	1·7% (1·0–2·8)	2·0% (2·0–2·0)	0·9% (0·5–1·3)
	Honduras (E)	0·9 (0·6–1·2)	1·6% (1·1–2·1)	94·7% (92·6–96·2)	1·3% (0·7–2·4)	0·0% (0·0–0·0)	0·1% (0·1–0·2)
	India (E)	118·6 (90·5–150·5)	6·6% (5·1–8·4)	61·5% (48·1–72·5)	28·4% (17·4–41·9)	1·1% (1·1–1·1)	0·3% (0·2–0·5)
	Indonesia (E)	42·5 (34·9–52·2)	28·5% (23·0–34·3)	46·0% (36·0–55·0)	17·95 (10·5–27·3)	5·8% (5·8–5·8)	0·2% (0·1–0·2)
	Iran (E)	9·1 (7·0–11·7)	21·4% (16·2–27·2)	73·9% (67·6–79·4)	0·0% (0·0–0·1)	26·5% (26·5–26·5)	0·0% (0·0–0·1)
	Iraq (F)	2·2 (1·5–2·9)	0·0% (0·0–0·0)	100·0% (100·0–100·0)	0·0% (0·0–0·0)	0·0% (0·0–0·0)	0·1% (0·1–0·2)
	Kazakhstan (F)	3·5 (2·4–4·8)	0·0% (0·0–0·0)	98·0% (96·2–99·0)	0·0% (0·0–0·0)	0·0% (0·0–0·0)	0·1% (0·1–0·1)
	Kenya (C)	86·5 (77·7–97·2)	53·6% (47·5–59·5)	25·5% (19·9–31·6)	14·8% (9·6–21·2)	5·1% (5·1–5·1)	1·7% (1·1–2·6)
	Kyrgyzstan (F)	0·1 (0·1–0·1)	40·6% (32·8–48·9)	59·4% (51·1–67·2)	0·0% (0·0–0·0)	0·1% (0·1–0·1)	0·0% (0·0–0·0)
	Laos (E)	11·4 (11·0–12·0)	84·9% (80·5–88·2)	12·5% (9·1–16·9)	1·4% (0·7–2·2)	18·3% (18·3–18·3)	1·2% (0·7–1·8)
	Liberia (C)	33·2 (30·5–36·1)	58·7% (53·9–63·8)	26·4% (22·0–30·8)	12·7% (8·6–17·9)	12·4% (12·4–12·4)	25·2% (17·1–34·9)
	Madagascar (C)	40·4 (39·5–41·5)	91·8% (89·3–93·7)	3·3% (2·4–4·3)	2·2% (1·4–3·3)	34·1% (34·1–34·1)	0·5% (0·3–0·7)
	Malawi (C)	81·8 (74·0–90·4)	52·2% (47·2–57·6)	35·7% (30·1–41·5)	9·9% (6·5–14·2)	10·0% (10·0–10·0)	18·2% (12·3–25·9)
	Malaysia (E)	44·3 (31·2–60·3)	1·9% (1·4–2·6)	92·6% (90·0–94·6)	1·0% (0·5–1·7)	16·2% (16·2–16·2)	0·6% (0·4–0·9)
	Mali (C)	64·0 (58·6–70·6)	61·1% (55·2–66·5)	22·6% (17·9–28·0)	15·9% (11·0–21·5)	18·1% (18·1–18·1)	10·2% (7·0–14·8)
	Mauritania (C)	6·2 (5·6–7·0)	56·5% (50·2–62·5)	33·5% (26·9–40·3)	8·5% (5·7–12·4)	18·7% (18·7–18·7)	2·6% (1·7–3·8)
	Mexico (E)	28·5 (19·8–40·1)	0·0% (0·0–0·0)	97·2% (95·3–98·4)	0·0% (0·0–0·0)	0·0% (0·0–0·0)	0·1% (0·1–0·1)
	Morocco (F)	5·1 (3·6–7·1)	0·0% (0·0–0·0)	98·5% (97·2–99·3)	0·0% (0·0–0·0)	0·0% (0·0–0·0)	0·2% (0·1–0·3)
	Mozambique (E)	131·4 (124·7–139·5)	75·4% (71·0–79·4)	19·7% (15·5–24·0)	4·3% (2·9–6·2)	14·5% (14·5–14·5)	14·5% (9·9–20·9)
	Myanmar (E)	68·4 (64·7–72·9)	79·5% (74·5–83·9)	14·5% (10·6–19·0)	6·0% (3·3–9·7)	18·1% (18·1–18·1)	1·6% (1·0–2·4)
	Namibia (E)	12·0 (9·5–15·4)	19·5% (15·0–24·4)	67·5% (62·1–72·5)	1·5% (1·0–2·3)	2·7% (2·7–2·7)	1·1% (0·8–1·5)
	Nepal (E)	1·8 (1·4–2·3)	22·6% (17·0–29·2)	71·1% (63·7–77·6)	1·1% (0·6–2·0)	0·3% (0·3–0·3)	0·5% (0·3–0·7)
	Nicaragua (E)	8·1 (7·3–9·1)	65·5% (58·4–72·5)	33·6% (26·5–40·8)	0·1% (0·1–0·2)	5·2% (5·2–5·2)	0·4% (0·3–0·6)
	Niger (C)	44·5 (34·6–57·2)	3·0% (2·3–3·8)	36·5% (26·3–47·2)	58·4% (47·1–69·6)	1·6% (1·6–1·6)	12·2% (8·2–17·7)
	Nigeria (C)	424·4 (366·4–499·2)	42·4% (35·8–48·8)	19·2% (14·1–25·3)	37·8% (28·9–47·2)	16·0% (16·0–16·0)	4·4% (2·8–6·6)
	North Korea (E)	2·1 (1·4–2·9)	4·0% (2·8–5·6)	94·0% (91·7–95·8)	1·7% (0·8–3·1)	1·4% (1·4–1·4)	0·2% (0·1–0·3)
	Oman (F)	2·7 (1·9–3·8)	0·0% (0·0–0·0)	98·1% (97·3–98·8)	0·0% (0·0–0·0)	..	0·1% (0·1–0·1)
	Pakistan (C)	22·5 (19·6–26·5)	53·6% (45·3–61·2)	22·6% (16·6–29·4)	22·8% (14·4–34·2)	1·9% (1·9–1·9)	0·3% (0·2–0·4)
	Panama (E)	7·4 (5·2–10·3)	0·0% (0·0–0·0)	97·5% (96·5–98·3)	0·0% (0·0–0·0)	0·0% (0·0–0·0)	0·3% (0·2–0·4)
	Papua New Guinea (C)	12·7 (11·3–14·4)	57·6% (50·8–64·6)	35·6% (28·3–43·3)	6·3% (4·0–9·1)	8·7% (8·7–8·7)	1·4 % (0·9–2·0)
	Paraguay (F)	6·1 (4·8–7·8)	26·4% (20·3–32·9)	69·6% (63·0–75·8)	0·0% (0·0–0·0)	12·4% (12·4–12·4)	0·4% (0·2–0·5)
	Peru (C)	7·1 (5·7–8·7)	33·4% (26·8–40·6)	60·6% (52·7–68·3)	3·0% (1·7–5·1)	7·8% (7·8–7·8)	0·1% (0·0–0·1)
	Philippines (E)	9·6 (8·1–11·5)	46·6% (38·5–55·0)	46·3% (38·1–55·1)	1·7% (0·9–2·9)	3·5% (3·5–3·5)	0·1% (0·1–0·2)
	Rwanda (C)	41·7 (40·0–44·2)	82·4% (77·8–86·0)	11·7% (8·7–15·1)	1·5% (1·0–2·2)	15·0% (15·0–15·0)	2·5% (1·6–3·7)
	São Tomé and Príncipe (E)	4·1 (3·8–4·4)	72·1% (66·9–77·1)	26·5% (21·3–31·8)	0·8% (0·5–1·1)	39·8% (39·8–39·8)	12·8% (8·9–17·7)
	Saudi Arabia (E)	38·7 (27·3–54·7)	0·0% (0·0–0·0)	93·5% (92·5–94·4)	0·0% (0·0–0·0)	..	0·1% (0·1–0·2)
	Senegal (C)	33·2 (31·9–34·8)	82·5% (78·7–85·9)	10·0% (7·6–13·3)	4·5% (3·0–6·5)	19·4% (19·4–19·4)	1·1% (0·7–1·6)
	Sierra Leone (C)	15·1 (12·7–17·9)	23·9% (20·0–28·2)	41·8 (32·6–50·2)	32·6% (23·6–43·3)	1·7% (1·7–1·7)	12·1% (8·1–17·6)
	Solomon Islands (E)	3·7 (3·4–4·1)	72·3% (64·8–78·2)	25·9% (19·7–33·4)	1·8% (1·1–2·7)	13·5% (13·5–13·5)	2·3% (1·6–3·5)
	Somalia (C)	0·7 (0·5–0·9)	20·4% (15·8–25·3)	31·5% (22·6–40·9)	47·6% (36·1–59·5)	0·2% (0·2–0·2)	0·7% (0·4–1·0)
	South Africa (E)	25·3 (18·5–34·1)	3·7% (2·7–4·9)	79·3% (76·1–82·4)	0·1% (0·0–0·1)	0·1% (0·1–0·1)	0·1% (0·1–0·2)
	South Korea (E)	1·1 (0·8–1·4)	0·0% (0·0–0·0)	91·7% (88·2–94·1)	5·5% (3·0–8·9)	..	0·0% (0·0–0·0)
	South Sudan (C)	28·3 (25·7–31·0)	61·9% (56·3–67·9)	28·6% (22·8–35·2)	7·8% (4·9–11·3)	15·8% (15·8–15·8)	2·7% (1·8–3·9)
	Sri Lanka (F)	2·6 (2·1–3·2)	36·9% (29·4–45·0)	60·8% (52·6–68·6)	0·0% (0·0–0·1)	2·0% (2·0–2·0)	0·1% (0·1–0·2)
	Sudan (C)	98·2 (88·1–110·4)	57·5% (51·0–63·9)	28·4% (21·8–36·0)	12·7% (7·9–18·5)	33·2% (33·2–33·2)	2·8% (1·8–4·1)
	Suriname (C)	1·5 (1·1–1·9)	22·2% (17·1–28·5)	71·3% (65·1–76·8)	0·2% (0·1–0·3)	22·2% (22·2–22·2)	0·8% (0·5–1·1)
	Syria (F)	0·4 (0·3–0·6)	0·0% (0·0–0·0)	98·6% (97·5–99·4)	0·0% (0·0–0·0)	0·0% (0·0–0·0)	0·1% (0·1–0·2)
	Tajikistan (F)	0·6 (0·5–0·8)	9·6% (7·2–12·8)	90·3% (87·1–92·7)	0·0% (0·0–0·0)	0·1% (0·1–0·1)	0·5% (0·3–0·7)
	Tanzania (C)	183·6 (168·5–200·4)	64·2% (58·7–69·8)	28·2% (22·8–34·0)	7·1% (4·7–10·4)	12·8% (12·8–12·8)	7·0% (4·6–10·0)
	Thailand (E)	19·6 (17·1–23·0)	55·2% (46·8–62·9)	40·1% (32·3–48·3)	0·8% (0·4–1·4)	24·5% (24·5–24·5)	0·1% (0·0–0·1)
	Timor-Leste (E)	4·9 (4·5–5·5)	69·2% (61·8–75·2)	29·8% (23·8–37·1)	0·5% (0·3–0·8)	15·1% (15·1–15·1)	2·3% (1·5–3·5)
	Togo (C)	16·1 (14·1–18·4)	42·6% (37·1–48·2)	31·3% (25·0–37·9)	22·8% (16·2–31·2)	15·3% (15·3–15·3)	7·7 % (5·0–11·3)
	Turkey (F)	19·5 (13·5–26·8)	0·0% (0·0–0·0)	98·1% (96·6–99·0)	0·0% (0·0–0·0)	0·0% (0·0–0·0)	0·1% (0·0–0·1)
	Turkmenistan (F)	1·1 (0·8–1·5)	0·0% (0·0–0·0)	98·2% (96·9–99·0)	0·0% (0·0–0·0)	0·0% (0·0–0·0)	0·2% (0·1–0·3)
	Uganda (C)	173·9 (162·6–187·9)	71·1% (65·7–75·9)	14·1% (10·9–17·9)	13·8% (8·9–19·5)	16·3% (16·3–16·3)	8·9% (5·9–12·8)
	Uzbekistan (F)	1·3 (0·9–1·7)	14·7% (10·8–19·2)	85·1% (80·6–89·1)	0·0% (0·0–0·0)	0·2% (0·2–0·2)	0·1% (0·1–0·1)
	Vanuatu (E)	0·7 (0·5–0·9)	26·6% (20·4–33·1)	70·7% (63·7–77·1)	1·1% (0·6–1·9)	2·7% (2·7–2·7)	3·1% (2·0–4·6)
	Venezuela (C)	11·2 (7·9–15·6)	0·8% (0·5–1·0)	81·1% (77·2–84·3)	3·2% (1·7–5·5)	11·3% (11·3–11·3)	0·2% (0·2–0·3)
	Vietnam (E)	12·4 (10·7–14·2)	55·7% (48·3–63·7)	40·1% (32·1–48·4)	3·9% (2·1–6·3)	2·3% (2·3–2·3)	0·1% (0·1–0·1)
	Yemen (C)	10·9 (9·3–13·1)	46·2% (38·3–53·6)	25·9% (19·1–33·7)	27·5% (18·5–38·4)	6·0% (6·0–6·0)	1·3% (0·8–1·9)
	Zambia (E)	119·5 (107·2–134·8)	54·2% (47·9–60·2)	40·5% (34·2–47·1)	3·3% (2·1–4·9)	13·8% (13·8–13·8)	12·2% (8·1–17·5)
	Zimbabwe (E)	43·3 (41·3–45·8)	81·2% (76·7–85·1)	13·0% (9·8–16·8)	2·2% (1·4–3·1)	11·0% (11·0–11·0)	0·7% (0·5–1·1)

Spending is reported in 2018 US$. Income groups are 2018 World Bank income groups. 95% uncertainty intervals are shown in parentheses. C=controlling. E=eliminating. F=malaria free.

**Figure 4:**
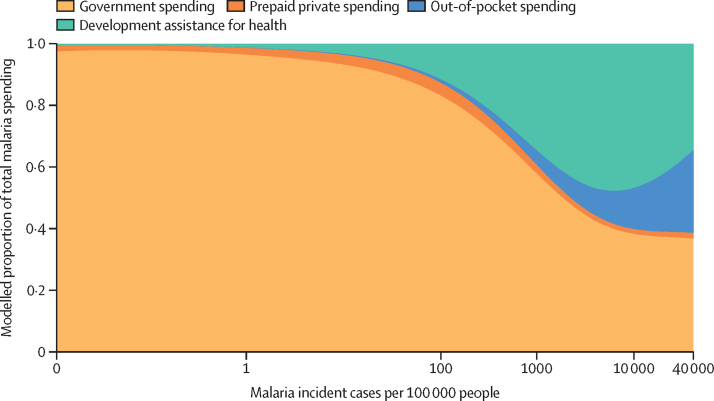
Composition of malaria spending by source and malaria cases per 100 000 people, 2016 Malaria incident cases from the Global Burden of Diseases, Injuries, and Risk Factors Study 2017.

To capture the variation in malaria spending, including within elimination status categories, figure 4 depicts 2016 malaria spending by source against malaria incidence per 100 000 people. As the incidence of malaria declines toward zero, government financing becomes by far the largest source of financing, a representation of the investments in prevention and surveillance by the government crucial to attaining malaria elimination and the lower levels of development assistance in these countries, which is tied to their higher income status. Countries with high malaria burden sourced financing from an array of sources, including development assistance, as well as the OOP spending incurred by people seeking treatment for malaria. Where people are most affected by malaria—as opposed to regions where elimination is in reach—malaria treatment, prevention, and control are financed predominately by households and donors (figure 4).

## Discussion

In 2016, $4·3 billion (95% UI 4·2–4·4) was spent on malaria worldwide. This is an 8·5% (95% UI 8·1–8·9) per year increase over malaria spending in 2000 ($1·2 billion, 1·0–1·3). Global government spending for malaria increased by 4·0% (3·8–4·2) and OOP spending by 3·8% (3·3–4·2), between 2000 and 2016. Overall, from 2000 to 2016, global financing of malaria activities shifted from government and OOP spending to a predominance of development assistance. The mix of financing sources, however, depended largely on the burden of malaria. In malaria control countries, where malaria incidence is highest, DAH and OOP spending were together larger than government expenditure in 2016. Where countries were actively in pursuit of elimination, governments financed the bulk of activities.

A major driver of the growth in malaria spending was the increase in development assistance for malaria. From 2000 to 2016, development assistance for malaria grew 18·0% annually on average. The growth in development assistance was fuelled by the emergence of new malaria leadership. Since 2000, three major initiatives have been launched: the US President's Malaria Initiative, the Global Fund, and Unitaid raised malaria on the global agenda and helped to mobilise billions in development assistance. The Bill & Melinda Gates Foundation has also invested substantially in malaria control and elimination.^5^ The USA has been the largest source of funding for malaria since 2008, contributing a total of $11·7 billion between 1990 and 2018 and $876 million in 2018 alone. In 2016, the largest shares of development assistance for malaria were disbursed through the Global Fund ($1·0 billion or 42·6%) and US bilateral aid agencies including the US President's Malaria Initiative ($374 million or 15·5%).

The development assistance mobilised by these initiatives and other malaria fundraising campaigns focused predominantly on prevention and control in 2016. Of total development assistance for malaria in 2016, 19·5% was disbursed for diagnosis and treatment. Development assistance for administrative costs and global or regional projects made up an additional 20·4%. This financing enhances frontline efforts to tackle malaria through global and regional coordination, which plays a role in improving treatment and prevention as well as more generally contributing to the global public good of advancing malaria elimination worldwide.

In countries where malaria burden is high, OOP spending is a major source of health financing. OOP spending on malaria, which is expended almost entirely on patient care, constituted 13·0% (95% UI 11·6–14·5) of all malaria spending globally—substantially higher than the OOP spending share of another high-priority disease area, HIV/AIDS, at 4·7% (1·9–8·6) in the 106 countries in our study in 2016.^28^ The OOP share of total health spending was 42·9% (41·8–43·9) in 2016 in the 106 countries, considerably higher than the OOP share of total health spending globally, at 19·4% (19·1–19·7). If we focus on the 47 control countries only, OOP spending is 19·0% (16·9–21·4) of total malaria spending, 6·5% (2·2–13·1) of HIV spending, and 43·5% (41·0–45·9) of total health spending. Nigeria and India—which have the first and third largest number of malaria incident cases per year worldwide—are above the 95th percentile in the share of total health expenditure financed OOP worldwide.^5^, ^29^ The role of OOP spending in health financing, particularly in malaria control countries, is concerning because more than 500 million people live below the poverty line in the low-income and lower-middle-income countries in sub-Saharan Africa and India where malaria predominately occurs.^22^ High OOP spending, particularly among poor people, probably translates into catastrophic health spending, adding financial woes to those already afflicted by malaria morbidity and mortality.

The most high-profile global action to reduce the costs of malaria treatment has focused on drug prices. The AMF-m, launched in 2009, aimed to make ACTs more affordable.^30^ Rigorous evidence has shown that the AMF-m reduced the OOP price of ACTs and improved access to ACTs in pilot countries.^31^, ^32^, ^33^ The reduction in drug costs is reflected in the only small increase in total OOP spending between 2000 and 2016, even as prevalence of treatment-seeking and ACT coverage rose considerably. The substantial increases in development assistance for malaria, and sustained government spending also ensured that malaria prevention interventions such as insecticide-treated bednets and indoor residual spraying were provided free of charge in many countries, precluding households from having to spend OOP.

Even so, the large OOP share of malaria spending raises the question of whether more should be done to reduce the OOP costs of malaria treatment. High OOP payments might deter people with malaria from accessing effective treatment in a timely manner. Such delays or avoidance of care can not only heighten risk of disability, complications, or death, but also contribute to onward transmission of malaria to others.^34^, ^35^ Reducing OOP spending on malaria presents an opportunity to improve equitable access to quality-assured treatment. This includes ensuring drugs are in stock in the public sector and that government-run health facilities are accessible at a low cost to patients. Such efforts also have the potential to improve financial risk protection, helping countries make progress toward universal health coverage. The small share of malaria spending financed by prepaid private sources underscores the potential to expand prepayment mechanisms that cover the financial costs of malaria treatment.

The large share of malaria spending sourced OOP might also be related to government financing. In 2016, government spending made up 28·2% (95% UI 27·1–29·3) of malaria expenditure. By contrast, in the 106 countries in our study, government spending as a share of HIV/AIDS expenditure was 60·1% (53·0–63·7) and as a share of total health spending was 51·0% (48·6–53·2) in 2016. Globally, government spending makes up 73·9% (73·3–74·6) of total health expenditure. The lower levels of government spending in the 106 countries in our study underscore that the structure of financing varies depending on the disease area, but also that in countries where malaria occurs, governments contribute a smaller share of health funding.

The low levels of public financing suggest raising additional public revenues might be an opportunity for more malaria financing. The mechanisms used to mobilise additional funds are not immediately apparent however. No upper-middle-income country is among the top 20 malaria burden countries. Countries with low gross domestic product per person face an array of difficulties in mobilising more government resources, including the low incomes of the population at large, low tax collection rates, and low governance capacity more generally. Corruption, a major issue in many malaria-endemic countries, can siphon public resources away from endeavours to improve social welfare like malaria control and elimination.^36^ Government resources can be reallocated to health and to malaria but this might require cutting other public budgets, with the resulting consequences for social welfare. Other opportunities to ensure resources go further include improving the efficiency of health systems, making the same amount of investment cover more people at risk or accentuate effect. Efficiency gains can be difficult to identify and realise in practice however. Over time and across countries, the effect of investments can vary substantially.

Overcoming these challenges to raising resources for malaria could result in major positive returns on investment. Research has shown that declines in malaria incidence have increased household per capita consumption.^37^ Other studies have established a connection between malaria and poverty.^38^ Major up-front investments in new technologies—such as malaria vaccines, new drugs, or gene drive—have the potential to dramatically reduce the resources required to combat malaria in the future, particularly if coordinated financing mechanisms can make these technologies affordable. Spending on malaria health system strengthening can support efforts to combat malaria while also having positive effects for the health system as a whole. However, research on malaria is not evenly distributed across malaria-endemic countries, potentially limiting the potential for innovation adapted to distinct country contexts.^39^ Purely in terms of public finance, governments in malaria-free and eliminating countries spent $79·8 less per capita on malaria in 2016 compared with control countries. Eliminating malaria might free up public resources to dedicate to other health problems.

Finally, our estimates showed how far spending is from malaria funding targets. In 2016, global spending on malaria fell more than $2 billion short of the $6·6 billion target set by WHO. Reaching that goal will involve a nearly 50% increase in malaria resources. Because we estimate OOP spending on malaria for the first time, we estimate a smaller gap than previous estimates.^40^ However, we note that OOP spending, by reducing access to treatment in some settings, might slow progress in malaria control and elimination instead of contributing to the global malaria goals tied to funding targets. Overall, sustaining the reductions in malaria incidence and seriously pursuing malaria elimination will require raising more resources from governments and development assistance partners. Making sure that a focus on elimination does not waver as few cases remain is also crucial to preventing resurgence and permanently eliminating the disease in countries.^41^

The limitations of tracking global spending on malaria relate predominantly to the characteristics of malaria spending data. The data are limited by the extent to which data producers and managers define malaria spending similarly, including the sources of these funds. Global guidelines have been developed to instruct health accountants and other data analysts in the categories to be used, but many tracking exercises are limited by the scope of primary data, including the budgets and expenditure tracking done by governments, non-governmental organisations, and private for-profit entities. The input data were in some cases contradictory or had incomplete underlying documentation. We applied estimates of treatment-seeking for fever from Battle and colleagues^19^ to malaria incident cases, requiring the assumption that treatment-seeking for malaria is similar to treatment-seeking for all fevers. Government spending data were limited by the few country-years capturing spending on patient care. Augmenting these estimates with patient care spending was thus a crucial improvement. Compared with government spending data, even fewer data on OOP and prepaid private spending were available. For OOP, we relied substantially on existing estimates of malaria treatment-seeking that could be affected by recall bias and incomplete reporting. Estimates of OOP costs of drugs are sensitive to whether samples were nationally representative and are particularly sparse, and thus required modelling for most country-years. The appendix (section S5) shows the number of points available for each component of the analysis. This highlights further the sparsity of data in some areas, notably drug costs and unit costs of malaria patient care. Additional data on the OOP and government costs of drugs and patient care would enhance the precision of our estimates. Overall, investments in primary data collection, notably outside of sub-Saharan Africa, on malaria treatment-seeking and costs would contribute to a better understanding of malaria spending globally.

Estimates of malaria spending globally and by source can provide insights crucial to putting countries on the path to malaria elimination. These estimates shed light on opportunities to make additional investments that could reduce transmission of malaria and alleviate catastrophic and impoverishing payments, helping countries pursue universal health coverage in addition to fighting malaria. More broadly, disease-specific spending estimates like these can be used to identify investments gaps and compare spending across countries, time, and disease burden, helping policy makers to make informed decisions about investments in health.
