# Supplementary material for: Tracking spending on malaria by source in 106 countries, 2000–16: an economic modelling study
Source: Lancet Infect Dis. 2019 Jul;19(7):703–16. doi: 10.1016/S1473-3099(19)30165-3 (PMC6595179; doi:10.1016/S1473-3099(19)30165-3)
Supplement: Supplementary appendix [file mmc1.pdf]

THE LANCET Infectious Diseases

Supplementary webappendix

This webappendix formed part of the original submission and has been peer reviewed. We post it as supplied by the authors.

Supplement to: Haakenstad A, Harle AC, Tsakalos G, et al. Tracking spending on malaria by source in 106 countries, 2000–16: an economic modelling studyl. *Lancet Infect Dis* 2019; published online April 25. http://dx.doi.org/10.1016/S1473-3099(19)30165-3.

Supplementary Methods Appendix

Tracking total spending on malaria by source in 106 countries, 2000-2016

Version: March 1, 2019

Table of contents

Section	Page
S1: Currency conversion	3
S2: Government health expenditure as a source on malaria	4
Data	4
Modeling government expenditure on malaria patient care	5
Modeling total government expenditure on malaria	10
S3: Out-of-pocket expenditure on malaria	13
Price-volume model	13
Modeling total out-of-pocket expenditure on malaria	18
S4: Prepaid private expenditure on malaria	19
S5: Malaria elimination status of the 106 countries	20
S6: Data density	23
S7: Probabilistic sensitivity analyses	25
S8: References	26

S1: Currency conversion

All malaria expenditure estimates were made and reported in 2018 United States dollars (USD). Data sources reported expenditure in either nominal local currency units (LCUs) or nominal USD. To convert nominal LCUs to USD, we applied deflators to nominal LCUs to inflate to 2018 LCUs. We then applied exchange rates to produce 2018 USD. When LCUs were not reported, we extracted reported expenditure in nominal USD, applied corresponding nominal exchange rates to produce nominal LCUs, inflated nominal LCUs to 2018 LCUs with deflators, and finally exchanged 2018 LCUs to 2018 USD. All deflators and exchange rates were extracted from the World Bank,[1] International Monetary Fund,[2] Penn World Tables,[3] the United Nations National Accounts[4] and the World Health Organization,[5] and were imputed to provide a complete series for each of the variables between 1950 and 2018. We then used several models including ordinary least-squares regression and mixed effects models, to complete each source series. More information about the approach to converters and deflators may be found in Global Burden of Disease Health Financing Collaborator Network (2019).[6]

S2: Government health expenditure as a source on malaria

Data

Government spending on malaria was drawn from three main sources. First, we extracted data on government spending reported by National Malaria Control Programmes (NMCPs) to the World Health Organization (WHO) and published in the WHO's annual World Malaria Reports (WMRs).[7] We conducted a web search of all existing National Health Accounts, including those hosted by the WHO on the Global Health Expenditure Database, and found 40 reports that estimated government expenditure as a source dedicated to malaria prevention, control, and treatment.[8] Finally, we extracted the government spending reported by governments submitting proposals and concept notes to the Global Fund. We excluded any projections for spending reported in these documents submitted to the Global Fund. Table 1 shows the number of countries-years available for each data source.

Table S1: Main data sources for government health expenditure as a source for malaria

Data source	Country-years of data
NMCP reports in the WMRs	890
NHAs	86
Global Fund proposals and concept notes	381

The spending reported by NMCPs to the WMRs typically capture expenditure on prevention activities, such as insecticide-treated nets, indoor residual spraying, and chemoprevention, as well as the purchase of antimalarial drugs and diagnostics. However, they do not include spending on patient care – e.g. spending on the labor, facilities, and others costs involved in providing care to malaria patients in government health facilities outside of drugs and diagnostics. Because the government spending numbers reported in the Global Fund concept notes and proposals are typically submitted by the same source – NMCPs – and aligned well with the reported numbers in the WMR, we assumed that this spending also did not include patient care.

Modeling government spending on patient care

To estimate government spending on patient care, we built a price-volume model focused on spending on inpatient and outpatient care, respectively, as shown in (1).

$$Gov_{mal\ patient\ care} = \quad (1)$$

$$(Gov\ cost\ per\ admission_{mal} * Public\ admissions_{mal}) +$$

$$(Gov\ cost\ per\ outpatient\ visit_{mal} * Public\ outpatient\ visits\ _{mal})$$

In (2) - (5) below, we show the equations used to estimate each element of $Gov_{mal\ patient\ care}$.

Estimates of $Gov\ cost\ per\ admission_{mal}$, shown in (2), were based on country-specific average $inpatient\ unit\ cost$ of all health conditions from Moses et al. (2018).[9] We extracted estimates of malaria inpatient unit costs for 22 country-years from 13 peer-reviewed articles, as shown in Table 2.[10-22] Only 11 country-years from these studies reported inpatient unit costs without spending on drugs and diagnostics. Therefore, we took the median share of non-drug, non-diagnostic spending and applied it to the remaining 11 country-years to generate $mal\ inpatient\ unit\ cost_{\text{non drug \& non diagnostic}}$. We converted these values to 2018 US dollars and took the ratio of these values to the inpatient unit costs from Moses et al. (2018) in the country and year in which the study took place. The median of this ratio (.69) was applied to all inpatient unit costs to calculate $Gov\ cost\ per\ admission_{mal}$.

$$Gov\ cost\ per\ admission_{mal} = \quad (2)$$

$$inpatient\ unit\ cost * med\ \left(\frac{mal\ inpatient\ unit\ cost_{\text{non drug \& non diagnostic}}}{inpatient\ unit\ cost}\right)$$

Table S2: Malaria inpatient unit costs extracted from peer-reviewed literature

Country	Year	Inpatient unit costs (2018 USD)	Non-drug, non-diagnostic inpatient unit costs (2018 USD)	Study
Bangladesh	2003-2005	60.9		Lubell et al. 2009
Cameroon	2013-2014	69.09	39.08	Maka et al. 2016
China	2014	926.17	175.11	Tang et al. 2017
Democratic Republic of the Congo	2005	37.97		Tsakala et al. 2005
Ghana	2009	29.37		Sicuri et al. 2013
India	1996-98	27.95	18.66	Gogtay et al. 2003
India	2003-2005	67.32		Lubell et al. 2009
Indonesia	2003-2005	72.25		Lubell et al. 2009
Kenya	2004-2005	151.86		Ayieko et al. 2009
Kenya	2015	86.51		Rakuomi et al. 2017
Kenya	2009	29.17		Sicuri et al. 2013
Myanmar	2003-2005	35.23		Lubell et al. 2009
Nigeria	2009	96.44	87.04	Lubell et al. 2011
Papua New Guinea	2007-2008		3.44	Davis et al. 2011
South Africa	2001	408.75		Muheki et al. 2004
Tanzania	2009	65.54	40.3	Lubell et al. 2011
Tanzania	2009	20.12		Sicuri et al. 2013
Thailand	2001	211.67	110.57	Kyaw et al. 2014
Uganda	2009	56.54	46.88	Lubell et al. 2011
Zimbabwe	2000	3120.64	2912.34	Hongoro and McPake 2003

$Public\ admissions_{mal}$, shown in (3), represents the number of inpatient admissions for malaria in government-run health facilities. NMCP programs reported the total number of $inpatient\ cases_{mal}$ to the WHO (not disaggregated by the public and private sector but capturing admissions in both sectors) and they are published in WMRs annually. Some missingness characterizes these reported values – malaria inpatient cases were available for 35% of country-years in our sample of 106 countries. For this reason, we modeled logit-transformed malaria admissions as a share of total admissions (from Moses et al. 2018) with spatiotemporal Gaussian process regression (ST-GPR) and the following covariates: malaria Lysenko 5 from the Global Burden of Disease (GBD) study 2017,[23] natural log transformed ten-year lag-distributed income per capita (LDI per capita), whether a country had a policy of providing artemisinin-based combination therapy (ACT) free-of-charge in the public sector as reported in WMRs, and random effects on country, region, and super-region. We back-transformed the dependent variable and multiplied the ratio with $inpatient\ cases_{mal}$ from Moses et al. (2018) to produce total malaria inpatient admissions for each of the 106 countries in our sample over 2000-2016.

$$Public\ admissions_{mal} = inpatient\ cases_{mal} * \left(\frac{public\ treated\ cases_{mal}}{treated\ cases_{mal}}\right) \quad (3)$$

$public\ treated\ cases_{mal}$ were based on estimates of treatment-seeking in the public sector among children under five with a fever in the last two weeks, as estimated by Battle et al. (2016).[24] $treated\ cases_{mal}$ were from the same source but focused on all treatment-seeking among children under five with a fever in the last two weeks. These are the only comprehensive set of estimates of malaria treatment-seeking available to-date. Furthermore, treatment-seeking rates for malaria among children have been shown to be similar to treatment-seeking among adults.[25,26]

To impute data for countries not included in the original study, we used a similar approach to Battle et al. (2016) – we modeled logit-transformed public treatment-seeking as a share of children under five with a fever in the last two weeks with ST-GPR and the following covariates: logit-transformed out of-pocket (OOP) expenditure as a share of total health expenditure from Global Burden of Disease Health Financing Collaborator Network (2019),[27] log LDI per capita, coverage of the diphtheria-tetanus-pertussis vaccine, four antenatal care visits, and skilled birth attendance, and the sociodemographic index (SDI),

all from the GBD 2017 study, and random effects on country, region, and super-region. $treated\ cases_{mal}$ were imputed with a linear mixed model with fixed effects on year and year squared and random effects on region and super-region. Since both $public\ treated\ cases_{mal}$ and $treated\ cases_{mal}$ were modeled as a share of fever cases, we multiplied these ratios by malaria incident cases from GBD 2017 to calculate counts of each measure. We assumed that the share of total admissions in the public sector is similar to the share of all treatment seeking that occurred in the public sector and thus apply the ratio $\frac{public\ treated\ cases_{mal}}{treated\ cases_{mal}}$ to $inpatient\ cases_{mal}$ to estimate $Public\ admissions_{mal}$.

Estimating $Gov\ cost\ per\ outpatient\ visit_{mal}$, shown in (4), deploys a similar approach to $Gov\ cost\ per\ admission_{mal}$ in (2). Estimates of $outpatient\ unit\ cost$ for all health conditions were sourced from Moses et al. (2018).[28] Malaria inpatient unit costs were extracted from 9 peer-reviewed articles.[29-37] Only 5 country-years reported outpatient unit costs without spending on drugs and diagnostics. The median share of non-drug, non-diagnostic spending was thus applied to the remaining 4 country-years studies to generate $mal\ outpatient\ unit\ cost_{\text{non drug \& non diagnostic}}$. We converted these values to 2018 US dollars and took the ratio of these values to the outpatient unit costs from Moses et al. (2018) in the country and year in which the study took place. The median of this ratio (.54) was applied to all outpatient unit costs to calculate $Gov\ cost\ per\ outpatient\ visit_{mal}$. Table 3 shows each of the point estimates extracted from peer reviewed literature.

$$Gov\ cost\ per\ outpatient\ visit_{mal} = \qquad (4)$$

$$outpatient\ unit\ cost * med\ \left(\frac{mal\ outpatient\ unit\ cost_{\text{non drug \& non diagnostic}}}{outpatient\ unit\ cost}\right)$$

Table S3: Malaria outpatient unit costs extracted from peer-reviewed literature

Country	Year	Outpatient unit costs (2018 USD)	Non-drug, non-diagnostic outpatient unit costs (2018 USD)	Study
China	2013-2014	1236.21		Liu et al. 2016
Ghana	2009	3.08		Sicuri et al. 2013
Kenya	2009	3.87		Sicuri et al. 2013
Nigeria	2013	24.38	20.11	Ezenduka et al. 2017
Nigeria	2016	15.57	1.60	Salwu et al. 2016
Papua New Guinea	2007-2008		0.517	Davis et al. 2011
South Africa	2004	27.01		Muheki et al. 2004
Tanzania	2003	2.55		Njau et al. 2008
Tanzania	2009	1.89		Sicuri et al. 2013
Tanzania	2005	6.64	5.51	Wiseman et a. 2006
Zambia	2005	6.99	1.81	Chanda et al. 2007

Estimates of $Public\ outpatient\ visits_{mal}$, shown in (5), was based on $Public\ admissions_{mal}$, estimated as in (3), and the $public\ treated\ cases_{mal}$ which is an input to those estimates. $Public\ admissions_{mal}$ were subtracted from $public\ treated\ cases_{mal}$ to calculate all outpatient visits for malaria in each country over 2000-2016.

$$Public\ outpatient\ visits_{mal} = public\ treated\ cases_{mal} - Public\ admissions_{mal} \quad (5)$$

Modeling total government spending on malaria

Total government spending is the sum of $Gov_{mal\ patient\ care}$ as described above and the government spending from the NMCP programs, as reported in WMRs and Global Fund proposals and concept notes ($NMCP\ Gov_{mal}$), appended by the government spending on malaria estimated in NHAs ($NHA\ Gov_{mal}$), as shown in (6).

$$Gov\ total_{mal} = NMCP\ Gov_{mal} + \ Gov_{mal\ patient\ care} \tag{6}$$
$$= NHA\ Gov_{mal}$$

To estimate a full time series for all years and countries in our study, we modelled $Gov\ total_{mal}$ as a share of total government spending excluding government spending on HIV with ST-GPR. We first considered the following covariates: malaria incidence, malaria prevalence, LDI per capita, coverage of indoor residual spraying (IRS), insecticide-treated nets (ITNs), and ACTs, the plasmodium falciparum parasite rate (PFPR), PFPR adjusted for IRS and ITNS, malaria Lysenko 1, 2 and 5 measures, one- and four-visit antenatal care coverage, the healthcare access and quality index (HAQI), the proportion of the population living in an urban area, coverage of skilled birth attendance (SBA), the sociodemographic index (SDI) and the universal health coverage (UHC) index. All the covariates measured as a proportion were logit-transformed. We sourced all covariate estimates from the GBD Study 2017 and from Malaria Atlas Project.

Because the availability of covariate data is higher for sub-Saharan Africa and patterns of incidence and intervention strategies differ from other regions, we split the government malaria spending data into two groups – sub-Saharan African and non-sub-Saharan African countries – and modeled each group separately. We performed covariate selection for each dataset, starting with the set of 19 potential covariates.

We first conducted a lasso regression to determine which covariates were least correlated, conditional on other covariates, with the fraction of government spending on malaria as the dependent variable. Covariates with an estimated coefficient of zero were removed from the set of possible covariates. We then used linear mixed effects regression to estimate all models including all possible combinations of the remaining covariates.

We then selected the intersection of 1000 best models with the lowest Akaike information criterion (AIC) and Bayesian information criterion (BIC) values. Finally, we completed a 10-fold cross-validation with out-of-sample predictions on these selected 1000 models. We selected the best model based on out-of-sample root mean squared error. The top-five models are shown in Table 4. Our final model is model 1 in both SSA and non-SSA sections.

Finally, we used a spatiotemporal Gaussian process regression (ST-GPR) to model government spending on malaria, independently modeling sub-Saharan African countries and non-sub-Saharan African countries. The first stage of ST-GPR was a mixed-effect model with random effects on Global Burden of Disease region and country, as well as the covariates selected using the method described above. To detect and reduce the influence of outlier data points, we used the selected model to measure Cooke's distance for each data point. We excluded each data point if Cooke's distance was greater than 4/n where n is the total number of $Gov\ total_{mal}$ data points.

Table S4: Top government spending models based on out-of-sample root-mean square error

SSA	Covariates	OOS RMSE
1	Malaria prevalence, ACT coverage, Malaria Lysenko PFPR (Highest Endemicity), Antenatal Care (4 visits) Coverage (proportion)	0.751537
2	Malaria prevalence, ACT coverage, Antenatal Care (4 visits) Coverage (proportion)	0.751913
3	Malaria prevalence, ACT coverage, Malaria Lysenko PFPR (Highest Endemicity), Antenatal Care (4 visits) Coverage (proportion), Healthcare access and quality index	0.752229
4	Malaria prevalence ACT coverage , Malaria Lysenko PFPR (Highest Endemicity), Antenatal Care (4 visits) Coverage (proportion) , Skilled Birth Attendance (proportion)	0.752298
5	Malaria prevalence , ACT coverage , Antenatal Care (4 visits) Coverage (proportion) , Healthcare access and quality index	0.752587
**Non - SSA**	**Covariates**	**OOS RMSE**
1	PFPR adjusted for ITN and IRS coverage, Malaria Lysenko PFPR (Epidemic), urbanicity, Skilled Birth Attendance (proportion)	0.824391
2	PFPR adjusted for ITN and IRS coverage, Malaria Lysenko PFPR (Epidemic), Skilled Birth Attendance (proportion)	0.824412
3	PFPR adjusted for ITN and IRS coverage, urbanicity, Skilled Birth Attendance (proportion)	0.824696

4	PFPR adjusted for ITN and IRS coverage, Skilled Birth Attendance (proportion)	0.824799
5	Malaria Lysenko PFPR (Epidemic), urbanicity, Skilled Birth Attendance (proportion)	0.824862

S3: Out-of-pocket expenditure on malaria

Price-volume model

We found only 55 points of malaria OOP expenditure, all sourced from NHAs. To augment this sparse dataset, we developed a price-volume model of malaria OOP, focused on the OOP costs of malaria treatment. Because of the substantial investment of governments and donors into malaria prevention and control, including financing of insecticide-treated nets, indoor residual spraying, community outreach and chemoprevention,[38] we assumed that the OOP spending on prevention is minimal, and focused on estimating the OOP spending on malaria treatment. Our price-volume model is shown in (7). The estimation strategy for each component of (7) is described in (8)-(12).

$$OOP_{mal} = \tag{7}$$

$$(OOP\ cost\ per\ admission_{mal} * Admissions_{mal}) +$$

$$(OOP\ cost\ per\ outpatient\ visit_{mal} * Outpatient\ visits_{\ mal}) +$$

$$OOP\ drug\ expenditure_{mal}$$

The approach to estimating volume of malaria treatment-seeking, both $Admissions_{mal}$ and $Outpatient\ visits_{\ mal}$, are described above. Briefly, inpatient cases were sourced from the WHO's WMRs and were modeled as share of all inpatient stays with ST-GPR. $Outpatient\ visits_{\ mal}$ are $treated\ cases_{mal}$, or treatment-seeking among children under five with a fever in the last two weeks based on Battle et al. (2016), minus $Admissions_{mal}$.

$OOP\ cost\ per\ admission_{mal}$, as shown in (8), was estimated similarly to $Gov\ cost\ per\ admission_{mal}$, with some modifications. Moses et al. (2018) was the source of $inpatient\ unit\ cost$. We adjusted these country-specific values to reflect the share of unit costs sourced OOP with $\frac{OOP\ inpatient\ spend}{Total\ inpatient\ spend}$, data extracted from 471 NHAs. $\frac{OOP\ inpatient\ spend}{Total\ inpatient\ spend}$ was logit-transformed and modeled with ST-GPR with the following covariates: logit-transformed OOP/THE from the Global Burden of Disease Financing Global Health Collaborator Network (2019), natural log-transformed LDI per capita and country, region,

and super-region effects. Estimates of OOP spending on malaria admissions ($mal\ OOP\ inpatient\ unit\ cost$) were extracted from nine peer-reviewed articles,[39-47] shown in Table S5, and converted to 2018 US dollars. To adjust average OOP inpatient unit costs to represent OOP costs per inpatient stay, we computed the median of the ratio $\frac{mal\ OOP\ inpatient\ unit\ cost}{\left(inpatient\ unit\ cost * \frac{OOP\ inpatient\ spend}{Total\ inpatient\ spend}\right)}$, which amounted to .30, and applied it to all other estimates of $inpatient\ unit\ cost * \frac{OOP\ inpatient\ spend}{Total\ inpatient\ spend}$ to generate $OOP\ cost\ per\ admission_{mal}$.

$$OOP\ cost\ per\ admission_{mal} = \tag{8}$$

$$inpatient\ unit\ cost * \frac{OOP\ inpatient\ spend}{Total\ inpatient\ spend} * med\left(\frac{mal\ OOP\ inpatient\ unit\ cost}{\left(inpatient\ unit\ cost * \frac{OOP\ inpatient\ spend}{Total\ inpatient\ spend}\right)}\right)$$

Table S5: Malaria OOP inpatient unit costs extracted from peer-reviewed literature

Country	Year	OOP malaria inpatient unit costs (2018 USD)	Study
China	2012	441.80	Xia et al. 2016
DRC	2011	217.19	Ilunga-Ilunga et al. 2015
Ethiopia	2003	67.49	Deressa et al. 2007
Ghana	2009	27.80	Sicuri et al. 2013
Kenya	2009	12.34	Sicuri et al. 2013
Malawi	2012	8.18	Hennessee et al. 2017
Mozambique	2001-2002	7.15	Castillo-Riquelme et al. 2008
Nigeria	2009	7.90	Onwujekwe O et al. 2013
South Africa	2001-2002	1.49	Castillo-Riquelme et al. 2008

Tanzania	2009	6.19	Sicuri et al. 2013
Uganda	2009	8.62	Nabyonga Orem et al. 2013
Zimbabwe	2014-2015	58.99	Gunda et al. 2017

Estimating $OOP\ cost\ per\ outpatient\ visit_{mal}$ employed a similar approach to strategy applied in (8). We used $outpatient\ unit\ cost$ from Moses et al. (2018) and adjusted those country-specific estimates to reflect the OOP unit costs of outpatient spending by applying the fraction of outpatient spending sourced OOP ($\frac{OOP\ outpatient\ spend}{Total\ outpatient\ spend}$). These data were extracted from 471 NHAs and were modeled with ST-GPR. As covariates, we used logit-transformed OOP/THE, log LDI per capita and random effects on country, region and super region. As shown in table 6, we extracted 13 country-years of data from 10 peer-reviewed articles to create $mal\ OOP\ outpatient\ unit\ cost_{\text{non drug}}$.[48-59] To adjust average OOP outpatient unit costs to represent malaria OOP non-drug unit costs, we computed the median of the ratio $\frac{mal\ OOP\ outpatient\ unit\ cost_{\text{non drug}}}{\left(outpatient\ unit\ cost * \frac{OOP\ outpatient\ spend}{Total\ outpatient\ spend}\right)}$, which amounted to .37, and applied it to all other estimates of $outpatient\ unit\ cost * \frac{OOP\ outpatient\ spend}{Total\ outpatient\ spend}$ to generate $OOP\ cost\ per\ outpatient\ visit_{mal}$.

$$OOP\ cost\ per\ outpatient\ visit_{mal} = \quad (9)$$

$$outpatient\ unit\ cost * \frac{OOP\ outpatient\ spend}{Total\ outpatient\ spend} * med\left(\frac{mal\ OOP\ outpatient\ unit\ cost_{\text{non drug}}}{\left(outpatient\ unit\ cost * \frac{OOP\ outpatient\ spend}{Total\ outpatient\ spend}\right)}\right)$$

Table S6: Malaria OOP non-drug outpatient unit costs extracted from peer-reviewed literature

Country	Year	OOP non-drug outpatient unit cost	Study
Burkina Faso	2000	22.84	Mugisha et al. 2002
China	2012	22.65	Xia et al. 2016
Kenya	2010	1.06	Chuma et al. 2010
Malawi	2009-2010	3	Ewing et al. 2011
Malawi	2004	6.48	Mota et al. 2009
Mozambique	2002	0.22	Castillo-Riquelme M et al. 2008
Nigeria	2013	0.34	Onwujekwe O et al. 2013
South Africa	2002	0.13	Castillo-Riquelme M et al. 2008
Sri Lanka	2004	3.65	Mustafa & Babiker 2007
Uganda	2012	0.03	Matovu et al. 2014
Uganda	2009	3.28	Nabyonga Orem et al. 2013
Vietnam	2004	3.13	Morel et al. 2008

We modeled drug expenditure on malaria broken down by spending on ACTs, which tend to be higher and have a different trend over 2000-2016, separately from other antimalarials, which included monotherapies, chloroquine, and other antimalarials, as shown in (10).

$$OOP\ drug\ expenditure_{mal} = ACT\ price * ACT\ covered\ cases + Other\ antimarial\ price * Other\ antimalarial\ covered\ cases \quad (10)$$

Data on OOP spending on ACTs and other antimalarials were sourced from reports on the outlet surveys conducted by ACTwatch (169),[60] Health Action International (738),[61] and as reported in a report by the Affordable Medicines Facility – malaria (AMFm).[62] The outlet surveys captured the price charged to

patients for an array of malaria drugs as well as their availability in private and public drug outlets, including government-run health facilities. For each outlet survey country-year, we took the average across the different ACTs and other antimalarials, respectively, weighted by their availability in different outlet types to approximate access to public and private OOP prices of drugs. All OOP drug prices were converted to 2018 USD. We modeled ACT and other antimalarial OOP prices separately.
Since 2000, the average cost of an antimalarial treatment course has decreased substantially,[63] influenced by negotiations to lower prices by AMF-m and the Global Fund. Furthermore, since 2000, many countries have instituted policies to provide ACTs free-of-charge in the public sector. Therefore, we modeled a country-specific trend for ACTs, incorporating changes to ACT-free policies.

Our main covariate for modeling OOP drug prices was the public procurement price of ACTs, sourced from the WHO's Global Price Reporting Mechanism (GPRM),[64] and the Global Fund's Price and Quality Reporting database,[65] which capture ACTs procured by NMCPs, non-governmental organizations and other institutions that received grants from the Global Fund or participate in the GPRM. For each country-year, we took the average procurement price paid for ACTs, weighted by the volume of drugs purchased, and converted all prices to 2018 US dollars. A full time series of ACT procurement prices was modeled with ST-GPR with log-transformed average global price of an ACT treatment course from Management Sciences for Health,[66] and random effects on country, region and super region. We log-transformed OOP ACT price, and applied a robust regression to log-transformed ACT procurement price, and an indicator for whether not a ACTs were provided free in the public sector in that country-year, as reported in the WHO's WMRs.

Average prices for other antimalarials have not changes substantially in real terms since 2000.[67] However, we wanted to capture regional and country variation in antimalarial OOP drug prices. We therefore modeled log-transformed other antimalarial OOP price with random effects on country, region and super-region with a linear mixed effects model.

The equations used for ACT covered cases and other antimalarial covered cases are shown in (11) and (12). We assumed that all treated cases for malaria received some sort of antimalarial drug. We relied on estimates of ACT coverage among children under five with a fever in the last two weeks estimated by the Bennett et al. (2017).[68] These data did not cover countries outside of sub-Saharan Africa, however, and so we imputed ACT coverage for these countries by replicating the same approach employed by

Bennet et al. (2017). We extracted the ACTs delivered for each country, as reported in the WHO's WMR. To fill in missing values in this dataset, we modeled log ACTs delivered per incident case in ST-GPR, with log LDI per capita as the sole covariate and random effects on country, region and super-region. Finally, logit-transformed ACT coverage was modeled with ST-GPR with log ACTs per capita and log LDI per capita and random effects on country, region, and super region. We multiplied ACT coverage and incidence cases to calculate $ACT\ treated\ cases$. We assume that all treated cases that did not get an ACT received another antimalarial. As described in the government spending section, $treated\ cases_{mal}$ were based on Battle et al. (2016) and $inpatient\ cases_{mal}$ were modeled based on the inpatient cases reported in the WHO's WMRs. We subtracted $inpatient\ cases_{mal}$ from $treated\ cases_{mal}$ to focus on non-inpatient treatment seeking.

$$ACT\ covered\ cases = \tag{11}$$

$$(treated\ cases_{mal} - inpatient\ cases_{mal}) * \frac{ACT\ treated\ cases}{treated\ cases_{mal}}$$

$$Other\ antimalarial\ covered\ cases = \tag{12}$$

$$(treated\ cases_{mal} - inpatient\ cases_{mal}) * \left(1 - \frac{ACT\ treated\ cases}{treated\ cases_{mal}}\right)$$

Modeling total out-of-pocket spending on malaria

We appended OOP_{mal} from the price-volume model to the 55 NHA points to create the full malaria OOP dataset. Using these data, we used ST-GPR to model logit-transformed OOP spending on malaria as a share of total OOP spending excluding OOP spending on HIV. The covariates for this model were LDI per capita, malaria incidence per capita, and the proportion of total OOP over total health expenditure, with random effects for country, region, and super-region.

S4: Prepaid private expenditure on malaria

Just 31 country-years of NHA data included prepaid private (PPP) expenditure on malaria. We combined these data with the estimates outlined above for OOP and government spending on malaria and malaria-focused development assistance for health (DAH) data from the Institute for Health Metrics and Evaluation to calculate total spending on malaria.[69] As a share of total spending on malaria, median PPP from the 31 NHA country-years was 1.5%. We computed the median ratio of malaria PPP share of non-PPP malaria spending to total PPP over non-PPP all health spending, as shown in (13), to calculate ρ (.36).

$$\rho = med \left[\frac{\left(\frac{PPP_{mal}}{DAH_{mal}+GHES_{mal}+OOP_{mal}} \right)}{\left(\frac{PPP}{DAH+GHES+OOP} \right)} \right] \tag{13}$$

We re-arranged terms and applied ρ to the estimated $DAH_{mal} + GHES_{mal} + OOP_{mal}$ and total health terms, as shown in (14).

$$PPP_{mal} = \rho * \left(\frac{PPP}{DAH+GHES+OOP} \right) * (DAH_{mal} + GHES_{mal} + OOP_{mal}) \tag{14}$$

S5: Malaria elimination status of the 106 countries

Table S7: Malaria elimination status by country

Elimination status in 2016	Countries
Control	Afghanistan<br>Angola<br>Benin<br>Bolivia<br>Brazil<br>Burkina Faso<br>Burundi<br>Cameroon<br>Central African Republic<br>Chad<br>Colombia<br>Congo<br>Cote d'Ivoire<br>Democratic Republic of the Congo<br>Djibouti<br>Equatorial Guinea<br>Eritrea<br>Ethiopia<br>Gabon<br>Ghana<br>Guinea<br>Guinea-Bissau<br>Guyana<br>Kenya<br>Liberia<br>Madagascar<br>Malawi<br>Mali<br>Mauritania<br>Niger<br>Nigeria<br>Pakistan<br>Papua New Guinea<br>Peru<br>Rwanda<br>Senegal<br>Sierra Leone<br>Somalia<br>South Sudan<br>Sudan<br>Suriname<br>Tanzania<br>The Gambia

	Togo<br>Uganda<br>Venezuela<br>Yemen
Eliminating	Bangladesh<br>Belize<br>Bhutan<br>Botswana<br>Cambodia<br>Cape Verde<br>China<br>Comoros<br>Costa Rica<br>Dominican Republic<br>Ecuador<br>El Salvador<br>Guatemala<br>Haiti<br>Honduras<br>India<br>Indonesia<br>Iran<br>Laos<br>Malaysia<br>Mexico<br>Mozambique<br>Myanmar<br>Namibia<br>Nepal<br>Nicaragua<br>North Korea<br>Panama<br>Philippines<br>Sao Tome and Principe<br>Saudi Arabia<br>Solomon Islands<br>South Africa<br>South Korea<br>Swaziland<br>Thailand<br>Timor-Leste<br>Vanuatu<br>Vietnam<br>Zambia<br>Zimbabwe
Malaria-free	Algeria<br>Argentina<br>Armenia

	Azerbaijan<br>Egypt<br>Georgia<br>Iraq<br>Kazakhstan<br>Kyrgyzstan<br>Morocco<br>Oman<br>Paraguay<br>Sri Lanka<br>Syria<br>Tajikistan<br>Turkey<br>Turkmenistan<br>Uzbekistan

S6: Data Density

Table S8: Availability of data by source and country elimination status

Source	Control	Eliminating	Malaria-Free	Total
ACTwatch	124	21		145
Affordable Medicine Facility - Malaria	127			127
Battle et al. (2016)	1598	1394	544	3536
Global Fund Concept Notes	73	55	6	134
Global Fund Price & Quality Reporting	3135	973	47	4155
Global Fund Proposals	131	81	12	224
Health Action International	207	21	1	229
Moses et al. (2018)	1598	1394	612	3604
NHA	120	29		149
WHO Global Price Reporting Mechanism	16074	2301	79	18454
WMR	2224	2367	690	5281
Total	25411	8636	1991	36038

Table S9: Availability of data by source and GBD super-region

Source	Central Europe, Eastern Europe, and Central Asia	High-income	Latin America and Caribbean	North Africa and Middle East	South Asia	Southeast Asia, East Asia, and Oceania	Sub-Saharan Africa	Total
ACTwatch						11	134	145
Affordable Medicine Facility - Malaria							127	127
Battle et al. (2016)	238	68	646	374	170	510	1530	3536

Global Fund Concept Notes	2		14	5	8	28	77	134
Global Fund Price & Quality Reporting	37		66	166	240	517	3129	4155
Global Fund Proposals	12		24	15	15	38	120	224
Health Action International			3	22	4	15	185	229
Moses et al. (2018)	272	68	646	408	170	510	1530	3604
NHAs				2		9	138	149
WHO Global Price Reporting Mechanism	69		604	917	254	1784	14826	18454
WMRs	317	96	1125	457	293	867	2126	5281
Total	947	232	3128	2366	1154	4289	23922	36038

S7: Probabilistic sensitivity analyses

We conducted probabilistic sensitivity analyses using the inputs with the most sparse data: unit costs of malaria inpatient stays and outpatient visits, drug prices and the ρ in the PPP model. We used the 25th and 75th percentile of each input and ran the analysis through for the spending category affected by the input. In Table S10, we show the results of these scenarios.

In the *Government* scenario, we ran the patient care component of the government spending model at the 25th and 75th percentiles of the outpatient and inpatient unit costs based on the values in the extracted literature shown in Tables S2 and S3. Once the 25th and 75th percentiles of government spending on patient care was computed based on these inputs, these estimates of patient care were added to the NMCP and Global Fund data, appended to the NHA data, and the whole dataset was modeled with ST-GPR to generate total government spending on malaria.

Two scenarios were assessed for OOP malaria spending. First, similar to the *Government* probabilistic sensitivity analyses, in the *OOP – patient care* scenario, we took the 25th and 75th percentiles of the outpatient and inpatient OOP unit costs, as shown in Tables S4 and S5, and ran the whole model through with these two values. In the *OOP – drug spending* scenario, we used the 25th and 75th percentiles of the OOP drug price data to, similarly, run through the full OOP model and calculate 25th to 75th percentile bounds on OOP spending.

Finally, in the PPP scenario, we calculated the 25th and 75th percentile of ρ, as shown in equation (13) based on the 31 NHA points and computed PPP based on equation (14) using the two different values.

Table S10: Estimates of government, OOP and PPP spending on malaria, using the 25th and 75th percentiles of select inputs for 2016

Scenario	Lower	Upper
Government	$ 962 million	$ 1,909 million
OOP – patient care	$ 281 million	$ 2,268 million
OOP – drug spending	$ 509 million	$ 647 million
PPP	$ 21 million	$ 288 million

S8: References

1. World Development Indicators (WDI) | Data Catalog. https://datacatalog.worldbank.org/dataset/world-development-indicators (accessed Jan 31, 2019).

2. World Economic Outlook Database October 2018. https://www.imf.org/external/pubs/ft/weo/2018/02/weodata/index.aspx (accessed Jan 31, 2019).

3. Robert C. Feenstra RI. Penn World Table 9.0. 2016. DOI:10.15141/S5J01T.

4. National Accounts - Analysis of Main Aggregates (AMA). https://unstats.un.org/unsd/snaama/Index (accessed Jan 31, 2019).

5. WHO. Global Health Observatory (GHO) data. WHO. http://www.who.int/gho/en/ (accessed Jan 31, 2019).

6. Global Burden of Disease Health Financing Collaborator Network. 2019. Past, present and future of global health financing: a review of country spending on health and development assistance for 195 countries, 1995-2050. *The Lancet*. Submitted Manuscript.

7. World Health Organization (WHO). World Malaria Report. WHO; Geneva, Switzerland. Available at: https://www.who.int/malaria/publications/world_malaria_report/en/ (Accessed January 15, 2019).

8. WHO. Global Health Expenditure Database: Documentation Centre: National Reports. WHO; Geneva, Switzerland. Available at: http://apps.who.int/nha/database/DocumentationCentre/Index/en (Accessed January 15, 2019.

9. Moses MW, Pedroza P, Baral R, Bloom S, Brown J, Chapin A, Compton K, Eldrenkamp E, Fullman N, Mumford JE, Nandakumar V, Rosettie K, Sadat N, Shonka T, Flaxman A, Vos T, Murray CJL, Weaver MR. 2018. Funding and services needed to achieve universal health coverage: applications of global, regional, and national estimates of utilisation of outpatient visits and inpatient admissions from 1990 to 2016, and unit costs from 1995 to 2016**.** *The Lancet Public Health*.

10. Ayieko P, Akumu AO, Griffiths UK, English M. 2009. The economic burden of inpatient paediatric care in Kenya: household and provider costs for treatment of pneumonia, malaria and meningitis. *Cost-Effectiveness and Resoure Allocation.* 7:3.

11. Davis WA, Clarke PM, Siba PM, Karunajeewa HA, Davy C, Mueller I, Davis TME. 2011. Cost-effectiveness of artemisinin combination therapy for uncomplicated malaria in children: data from Papua New Guinea. *Bulletin of the World Health Organization*. 89(3):211-220.

12. Gogtay NJ, Kadam VS, Desai S, Kamtekar KD, Dalvi SS, Kshirsagar NA. 2003. A cost-effectiveness analysis of three antimalarial treatments for acute, uncomplicated Plasmodium falciparum malaria in Mumbai, India. *Journal of Association of Physicians of India.* 51:877-879.

13. Hongoro C, McPake B. 2003. Hospital costs of high-burden diseases: malaria and pulmonary tuberculosis in a high HIV prevalence context in Zimbabwe. *Tropical Medicine and International Health.* 8(3):242-50.

14. Kyaw SS, Drake T, Ruangveerayuth R, Chierakul W, White NJ, Newton PN, Lubell Y. 2014. Cost of treating inpatient falciparum malaria on the Thai-Myanmar border. *Malaria Journal.* 13:416.

15. Lubell Y, Riewpaiboon A, Dondorp AM, von Seidlein L, Mokuolu OA, Nansumba M, Gesase S, Kent A, Mtove G, Olaosebikan R *et al*. 2011. Cost-effectiveness of parenteral artesunate for treating children with severe malaria in sub-Saharan Africa. *Bulletin of the World Health Organization*. 89(7):504-512.

16. Maka DE, Chiabi A, Obadeyi B, Mah E, Nguefack S, Nana P, Mbacham W, Mbonda E. 2016. Economic evaluation of artesunate and three quinine regimens in the treatment of severe malariain children at the Ebolowa Regional Hospital-Cameroon: a cost analysis. *Malaria Journal.* 15(1):587.

17. Muheki C, McIntyre D, Barnes KI. 2004. Artemisinin-based combination therapy reduces expenditure on malaria treatment in KwaZulu Natal, South Africa. *Tropical Medicine & International Health.* 9(9):959-966.

18. Rakuomi V, Okalebo F, Ndwigah S, Mbugua L. 2017. Cost effectiveness of pre-referral antimalarial treatment in severe malaria among children in sub-Saharan Africa. *Cost-effectiveness and Resource Allocation.* 15: 14.

19. Sicuri E, Vieta A, Lindner L, Constenla D, Sauboin C. 2013. The economic costs of malaria in children in three sub-Saharan countries: Ghana, Tanzania and Kenya. *Malaria Journal.* 12:307.

20. Svihrova V, Szilagyiova M, Krkoska D, Simekova K, Hudeckova H, Avdicova M. 2009. Analysis of the direct and indirect costs of treatment of imported malaria in the Slovak Republic. *Revista de Sociedade Brasiliera de Medicina Tropical.* 42(4):377-80.

21. Tang S, Feng D, Wang R, Ghose B, Hu T, Ji L, Wu T, Fu H, Huang Y, Feng Z. 2017. Economic burden of malaria inpatients during National Malaria Elimination Programme: estimation of hospitalization cost and its inter-province variation. *Malaria Journal.* 16(1):291. d

22. Tsakala TM, Tona GL, Mesia K, Mboma JC, Vangu JM, Voso SM, Kanja GL, Kodondi KK, Mabela M, Walo R. 2005. [Evaluation of prescriptions for inpatient treatment of malaria and gastroenteritis: Bondeko and St Joseph hospitals in Kinshasa]. Sante. 15(2):119-24.

23. James SL, Abate D, Abate KH, *et al.* Global, regional, and national incidence, prevalence, and years lived with disability for 354 diseases and injuries for 195 countries and territories, 1990–2017: a systematic analysis for the Global Burden of Disease Study 2017. *The Lancet* 2018; 392: 1789–858.

24. Battle KE, Bisanzio D, Gibson HS, Bhatt S, Cameron E, Weiss DJ, Mappin B, Dalrymple U, Howes RE, Hay SI, Gething PW. 2016. Treatment-seeking rates in malaria endemic countries. *Malaria Journal.* 15(20).

25. WHO. 2008. World malaria report 2008. WHO; Geneva, Switzerland. p. 215.

26. Deressa W. 2007. Treatment-seeking behaviour for febrile illness in an area of seasonal malaria transmission in rural Ethiopia. *Malaria Journal.* 6:49.

27. The Global Burden of Disease Financing Global Health Collaborator Network. 2019. Past, present, and future of global health financing: country spending, and development assistance for health for 195 countries, 1995–2050. *The Lancet.* Submitted Manuscript.

28. Moses MW, Pedroza P, Baral R, Bloom S, Brown J, Chapin A, Compton K, Eldrenkamp E, Fullman N, Mumford JE, Nandakumar V, Rosettie K, Sadat N, Shonka T, Flaxman A, Vos T, Murray CJL, Weaver MR. 2018. Funding and services needed to achieve universal health coverage: applications of global, regional, and national estimates of utilisation of outpatient visits and inpatient admissions from 1990 to 2016, and unit costs from 1995 to 2016**.** *The Lancet Public Health*.

29. Chanda P, Masiye F, Chitah BM, Sipilanyambe N, Hawela M, Banda P, Okorosobo T. 2007. A cost-effectiveness analysis of artemether lumefantrine for treatment of uncomplicated malaria in Zambia. *Malaria Journal.* 6.

30. Davis WA, Clarke PM, Siba PM, Karunajeewa HA, Davy C, Mueller I, Davis TME. 2011. Cost-effectiveness of artemisinin combination therapy for uncomplicated malaria in children: data from Papua New Guinea. *Bulletin of the World Health Organization*. 89(3):211-220.

31. Ezenduka CC, Falleiros DR, Godman BB. 2017. Evaluating the Treatment Costs for Uncomplicated Malaria at a Public Healthcare Facility in Nigeria and the Implications. *Pharmacoeconomics.* 1(3):185-194.

32. Liu Y, Zhang YL, Gao LJ, Qian D, Yang CY, Zhou RM, Xu BL, Zhang HW. 2016. Medical Costs on Malaria in Henan Province and Analysis of the Influential Factors. *Zhongguo Ji Sheng Chong Xue Yu Ji Sheng Chong Bing Za Zhi.* (1):11-7.

33. Muheki C, McIntyre D, Barnes KI. 2004. Artemisinin-based combination therapy reduces expenditure on malaria treatment in KwaZulu Natal, South Africa. *Tropical Medicine & International Health.* 9(9):959-966.

34. Njau JD, Goodman CA, Kachur SP, Mulligan J, Munkondya JS, McHomvu N, Abdulla S, Bloland P, Mills A. 2008. The costs of introducing artemisinin-based combination therapy: evidence from district-wide implementation in rural Tanzania. *Malaria Journal.* 7.

35. Salawu AT, Fawole OI, Dairo MD. 2016. Patronage and Cost of Malaria Treatment in Private Hospitals in Ibadan North L.G.A. South Western Nigeria. *Analysis of Ibadan Post-graduate Medicine.* 14(2):81-84.

36. Sicuri E, Vieta A, Lindner L, Constenla D, Sauboin C. 2013. The economic costs of malaria in children in three sub-Saharan countries: Ghana, Tanzania and Kenya. *Malaria Journal.* 12:307.

37. Wiseman V, Kim M, Mutabingwa TK, Whitty CJM. 2006. Cost-effectiveness study of three antimalarial drug combinations in Tanzania. *Plos Medicine.* 3:1844-1850.

38. Institute for Health Metrics and Evaluation (IHME). 2018. Financing Global Health 2017:Funding Universal Health Coverage and the Unfinished HIV/AIDS Agenda. IHME; Seattle, WA. Available at: http://www.healthdata.org/policy-report/financing-global-health-2017 (Accessed January 16, 2019).

39. Castillo-Riquelme M, McIntyre D, Barnes K. 2008. Household burden of malaria in South Africa and Mozambique: is there a catastrophic impact? *Tropical Medicine and International Health.* 13:108–122.

40. Deressa W, Hailemariam D, Ali A. 2007. Economic costs of epidemic malaria to households in rural Ethiopia. *Tropical Medicine and International Health* 12(10):1148-56.

41. Gunda R, Shamu S, Chimbari MJ, Mukaratirwa S. 2017.Economic burden of malaria on rural households in Gwanda district, Zimbabwe. *African Journal of Primary Health Care and Family Medicine.* 9(1):e1-e6.

42. Hennessee I, Chinkhumba J, Briggs-Hagen M, Bauleni A, Shah MP, Chalira A, Moyo D, Dodoli W, Luhanga M, Sande J, Ali D, Gutman J, Lindblade KA, Njau J, Mathanga DP. 2017. Household costs among patients hospitalized with malaria: evidence from a national survey in Malawi, 2012. *Malaria Journal.* 16(1):395. doi: 10.1186/s12936-017-2038-y.

43. Ilunga-Ilunga F, Levêque A, Laokri S, Dramaix M. 2015. Incidence of catastrophic health expenditures for households: an example of medical attention for the treatment of severe childhood malaria in Kinshasa reference hospitals, Democratic Republic of Congo. *Journal of Infection and Public Health.* 8:136–144.

44. Nabyonga Orem J, Mugisha F, Okui AP, Musango L, Kirigia JM. 2013. Health care seeking patterns and determinants of out-of-pocket expenditure for malaria for the children under-five in Uganda. *Malaria Journal.* 12:175.

45. Onwujekwe O, Uguru N, Etiaba E, Chikezie I, Uzochukwu B, Adjagba A. 2013. The economic burden of malaria on households and the health system in Enugu State southeast Nigeria. *PLoS One.* 8(11):e78362.

46. Sicuri E, Vieta A, Lindner L, Constenla D, Sauboin C. 2013.The economic costs of malaria in children in three sub-Saharan countries: Ghana, Tanzania and Kenya. *Malaria Journal.* 12:307.

47. Xia S, Ma JX, Wang DQ, Li SZ, Rollinson D, Zhou SS, Zhou XN. 2016. Economic cost analysis of malaria case management at the household level during the malaria elimination phase in The People's Republic of China. *Infectious Diseases of Poverty.* 5(1):50.

48. Castellani J, Mihaylova B, Siribié M, Gansane Z, Ouerdraogo AZ, Fouque F, Sirima SB, Evers SMAA, Paulus ATG, Gomes M. 2018. Household costs and time to treatment for children with severe febrile illness in rural Burkina Faso: the role of rectal artesunate. *Malaria Journal.* 17: 380.

49. Castillo-Riquelme M, McIntyre D, Barnes K. 2008. Household burden of malaria in South Africa and Mozambique: is there a catastrophic impact? *Tropical Medicine and International Health.* 1:108-22.

50. Chuma J, Okungu V, Molyneux C. 2010. The economic costs of malaria in four Kenyan districts: do household costs differ by disease endemicity? *Malaria Journal.* 2 (9): 149.

51. Ewing VL, Lalloo DG, Phiri KS, Roca-Feltrer A, Mangham LJ, SanJoaquin MA. 2011. Seasonal and geographic differences in treatment-seeking and household cost of febrile illness among children in Malawi. *Malaria Journal.* 10:32.

52. Matovu F, Nanyiti A, Rutebemberwa E. 2014. Household health care-seeking costs: experiences from a randomized, controlled trial of community-based malaria and pneumonia treatment among under-fives in eastern Uganda. *Malaria Journal.* 13:222.

53. Morel CM, Thang ND, Xa NX, Hung le X, Thuan le K, Van Ky P, Erhart A, Mills AJ, D'Alessandro U. 2008. The economic burden of malaria on the household in south-central Vietnam. *Malaria Journal.* 7:166.

54. Mota RE, Lara AM, Kunkwenzu ED, Lalloo DG. 2009. Health seeking behavior after fever onset in a malaria-endemic area of Malawi. *American Journal of Tropical Medicine and Hygiene.* 81(6):935-43.

55. Mugisha F, Kouyate B, Gbangou A, Sauerborn R. 2002. Examining out-of-pocket expenditure on health care in Nouna, Burkina Faso: implications for health policy. *Tropical Medicine and International Health.*7(2):187-96.

56. Mustafa MH, Babiker MA. 2007. Economic cost of malaria on households during a transmission season in Khartoum State, Sudan. *Eastern Mediterranean Health Journal.* 13(6):1298-307.

57. Nabyonga Orem J, Mugisha F, Okui AP, Musango L, Kirigia JM. 2013. Health care seeking patterns and determinants of out-of-pocket expenditure for malaria for the children under-five in Uganda. *Malaria Journal.* 12:175.

58. Onwujekwe O, Uguru N, Etiaba E, Chikezie I, Uzochukwu B, Adjagba A. 2013.The economic burden of malaria on households and the health system in Enugu State southeast Nigeria. *PLoS One.* 8(11):e78362.

59. Xia S, Ma JX, Wang DQ, Li SZ, Rollinson D, Zhou SS, Zhou XN. 2016. Economic cost analysis of malaria case management at the household level during the malaria elimination phase in The People's Republic of China. *Infectious Disease of Poverty.* 5(1):50.

60. ACTwatch. News & Publications. Available at: http://www.actwatch.info/publications (Accessed January 16, 2019).

61. Health Action International (HAI). Price & Availability Data: Database of medicine prices, availability, affordability and price components. HAI. Available at: http://haiweb.org/what-we-do/price-availability-affordability/price-availability-data/ (Accessed January 16, 2019).

62. Arnold F, Ye Y, Ren R, Yoder S, Hanson K, Goodman C, Tougher S, Mann A, Willey B. 2012. Independent Evaluation of Phase 1 of the Affordable Medicines Facility - malaria (AMFm): multi-country independent evaluation report: final report. *The Global Fund to Fight AIDS, Tuberculosis and Malaria.* Available at: https://unitaid.org/assets/Mid-term-evaluation-Affordable-medicines-for-malaria-facility-AMFm-Phase-1.pdf. (Accessed January 18, 2019).

63. Management Sciences for Health (MSH). 2018. International Medical Products Price Guide. Available at: http://mshpriceguide.org/en/home/ (Accessed January 16, 2019).

64. WHO. Global Price Reporting Mechanism for HIV, tuberculosis and malaria. WHO; Geneva, Switzerland. Available at: https://www.who.int/hiv/amds/gprm/en/ (Accessed January 16, 2019).

65. The Global Fund to Fight AIDS, Tuberculosis and Malaria. Price & Quality Reporting. The Global Fund; Geneva, Switzerland. Available at: https://www.theglobalfund.org/en/sourcing-management/price-quality-reporting/ (Accessed January 16, 2019).

66. Management Sciences for Health (MSH). 2018. International Medical Products Price Guide. Available at: http://mshpriceguide.org/en/home/ (Accessed January 16, 2019).

67. Based on an analysis of: Management Sciences for Health (MSH). 2018. International Medical Products Price Guide. Available at: http://mshpriceguide.org/en/home/ (Accessed January 16, 2019).

68. Bennett A, Bisanzio D, Yukich JO, Mappin B, Fergus CA, Lynch M, Cibulskis RE, Bhatt S, Weiss DJ, Cameron E, Gething PW, Eisele TP. 2017. Population coverage of artemisinin-based combination treatment in children younger than 5 years with fever and Plasmodium falciparum infection in Africa, 2003–2015: a modelling study using data from national surveys. *The Lancet Global Health.* 5:e418-27.

69. Global Burden of Disease Health Financing Collaborator Network. Past, present and future of global health financing: a review of country spending on health and development assistance for 195 countries, 1995-2050. *The Lancet*; Submitted Manuscript.
